# Supramolecular axial chirality in [N–I–N]^+^-type halogen bonded dimers†

**DOI:** 10.1039/d3sc03170e

**Published:** 2023-08-29

**Authors:** Shuguo An, Aiyou Hao, Pengyao Xing

**Affiliations:** a Key Laboratory of Colloid and Interface Chemistry of Ministry of Education and School of Chemistry and Chemical Engineering, Shandong University Jinan 250100 People's Republic of China xingpengyao@sdu.edu.cn

## Abstract

Axial chiral molecules are extensively used as skeletons in ligands for asymmetric catalysis and as building blocks of chiroptical materials. Designing axial chirality at the supramolecular level potentially endows a material with dynamic tunability and adaptivity. In this work, for the first time, we have reported a series of halogen-bonded dimeric complexes with axial chirality that were formed by noncovalent bonds. The [N–I–N]^+^-type halogen bond is highly directional and freely rotatable with good linearity and ultra-high bond energy; this bond was introduced to couple quinoline moieties with chiral substitutes. The resultant dimers were stable in solutions with thermo-resistance. Prominent steric effects from the 2′ chiral pendant allowed the chirality to be transferred to aryl skeletons with induced preferred axial chirality and optical activities. Halogen-bonded complexation presented visible emissions to afford luminescent axial chiral materials, whereby circularly polarized fluorescence and phosphorescence were achieved. The [N–I–N]^+^-type halogen bond performed as a powerful tool to construct functional axial chiral compounds, enriching the toolbox for asymmetric synthesis and optics.

## Abstract 2

The [N–I–N]^+^-type halogen bond performed as a powerful tool for the construction of functional axial chiral compounds, enriching the toolbox for asymmetric synthesis and optics.

## Graphical abstract

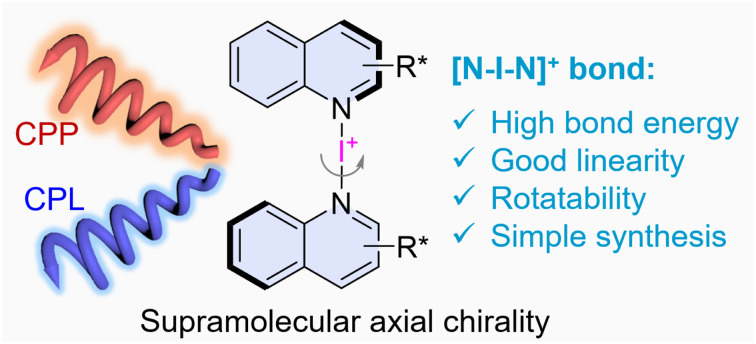

## Introduction

Being a basic feature of natural products, chirality expresses hierarchy across molecular to supramolecular and macroscopic levels.^1–3^ Molecular-level chirality represents a property that a molecule cannot be superimposed with its mirror entity. In contrast, the asymmetric packing of molecules oriented by noncovalent forces in the self-assembly exhibits chirality at the supramolecular scale, which correlates to the generation of versatile chiral nano/microarchitectures and other self-assemblies of diverse dimensions and sizes.^4^ Compared to the synthetic chemistry that produces chiral molecules by asymmetric catalysis or resolution, the supramolecular protocol is efficient as it works by assembling chiral molecules or through symmetry breaking with subsequent chiral amplification.^5–7^ The various noncovalent forces allow flexible design and fabrication of chiral-assembled structures towards advanced functions and applications for asymmetric sensing, recognition, resolution, and chiroptical materials. A state-of-the-art understanding of supramolecular chirality, however, still remains obscure due to the lack of classification, which is in sharp contrast to explicit molecular chirality comprising central, axial, planar, helical, and distorted types.^1^ A good example is the precise description of 2_1_ or other types of packing of planar aromatics, such as the supramolecular tilt chirality that depends on the X-ray structures.^8,9^ However, it is still limited to specific packings, such as the Sohncke space groups.^10^ It is hard to rationally describe supramolecular chirality in most cases. At the molecular level, axial chirality is generated when two pairs of substitutes are within a nonpolar orientation around an aryl–aryl bond, which limits the free rotation by the steric hindrance. Axial chirality commonly occurs in derivatives of allenes and biphenyl atropisomers, such as BINAP, where it has been widely utilized in asymmetric catalytic ligands and chiroptical applications.^11–15^ However, supramolecular interaction has not been applied to fabricate axial assemblies yet.

A halogen bond (or X-bond) is defined as the net attractive interaction between the nucleophilic and electrophilic regions of halogens.^16–19^ Halogen bonds, including I–O, Cl–O, Br–O, Cl–S, Cl–N, and Br–π interactions, have been found in protein complexes, which influence their chiral folding and functions.^20–23^ In many self-assembled systems containing halogens, halogen bonds play vital roles in tailoring and manipulating supramolecular chirality and optics.^24,25^ Nevertheless, the weak nature of halogen bonds (interaction energies are normally lower than 100 kJ mol^−1^) suffers from solvation and competitive electrophilic or nucleophilic agents, and consequently, it is challenging to achieve stable halogen-bonded supramolecular complexes in solution.^19,23,26–30^ The ionic I^+^ with two enhanced electrophilic regions, namely p-holes, could generate [N–I–N]^+^-type ionic halogen bonds with extremely boosted interaction energies of >180 kJ mol^−1^. These have emerged recently as a promising driving force to construct functional entities due to high stability and bond energy.^31–34^ For example, [N–I–N]^+^-type ionic halogen bonds have been employed to fabricate supramolecular frameworks, helical polymers, and other functional assemblies with finely tailored geometries.^20,35–38^ [N–I–N]^+^-type halogen bonding can be perceived as a bond with three centers and four electrons that is established by the stabilization of I^+^ through interaction with two Lewis bases; it involves two N–I secondary bonds. Should any of these bonds be compromised, it initiates the breakdown of the entire halogen bond.^33^ Hence, the binding constants for [N–I–N]^+^-type halogen bonding complexes cannot be determined using titration experiments in combination with relevant formulae^39^ or through Pall Thordarson's webpage,^40^ as is feasible with conventional host–guest complexes. Furthermore, solvents play a critical role. For instance, dimethyl formamide and acetone, containing carbonyl oxygen atoms that function as Lewis bases, can compete with quinoline derivatives for interaction with I^+^, potentially leading to the disruption of halogen bonding. This consideration also accounts for why past studies have often favored the use of dichloromethane or chloroform.^28,31,35,38^

**Scheme 1:**
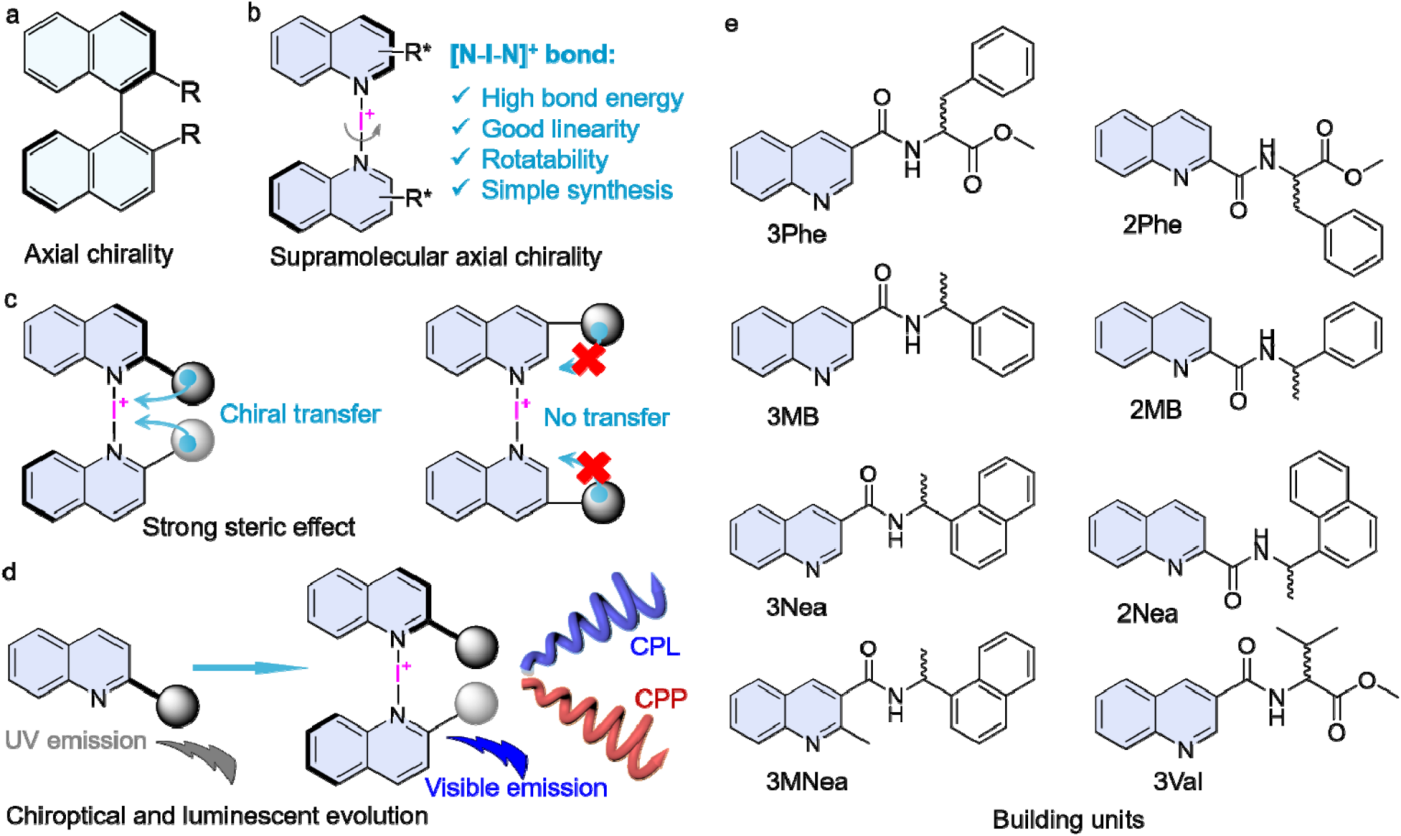
(a and b) Comparison of conventional and supramolecular axial chirality. (c) Strong steric effects. (d) Luminescent and chiroptical evolution by halogen bonding. (e) Chemical structures of the monomers.

The [N–I–N]^+^ bond is highly directional, with an N–I–N angle of ∼180°.^34^ This linearity inspired us to consider it as a rotational bond to couple two aromatics to create a pro-axial chiral complex.^41^ A chiral pendant that is conjugated adjacent to the [N–I–N]^+^ bond might direct the screw sense to give aryl axial chirality. To achieve this, we designed a series of quinoline building units with a chiral pendant at 2′ and 3′ positions (Scheme 1). An Ag(i) coordination-iodine substitution procedure was conducted to synthesize [N–I–N]^+^-bonded dimeric complexes with tailored substitutions. Experimental and computational results provided evidence that axial chirality was achieved with prominent steric effects. The supramolecular axial chirality showed resistance to solvation and thermal treatment. In addition, halogen-bonded complexation initiated luminescent evolution from the UV to the visible region, which led to the realization of circularly polarized fluorescence (CPL) and phosphorescence (CPP).

## Results and discussion

**Fig. 1:**
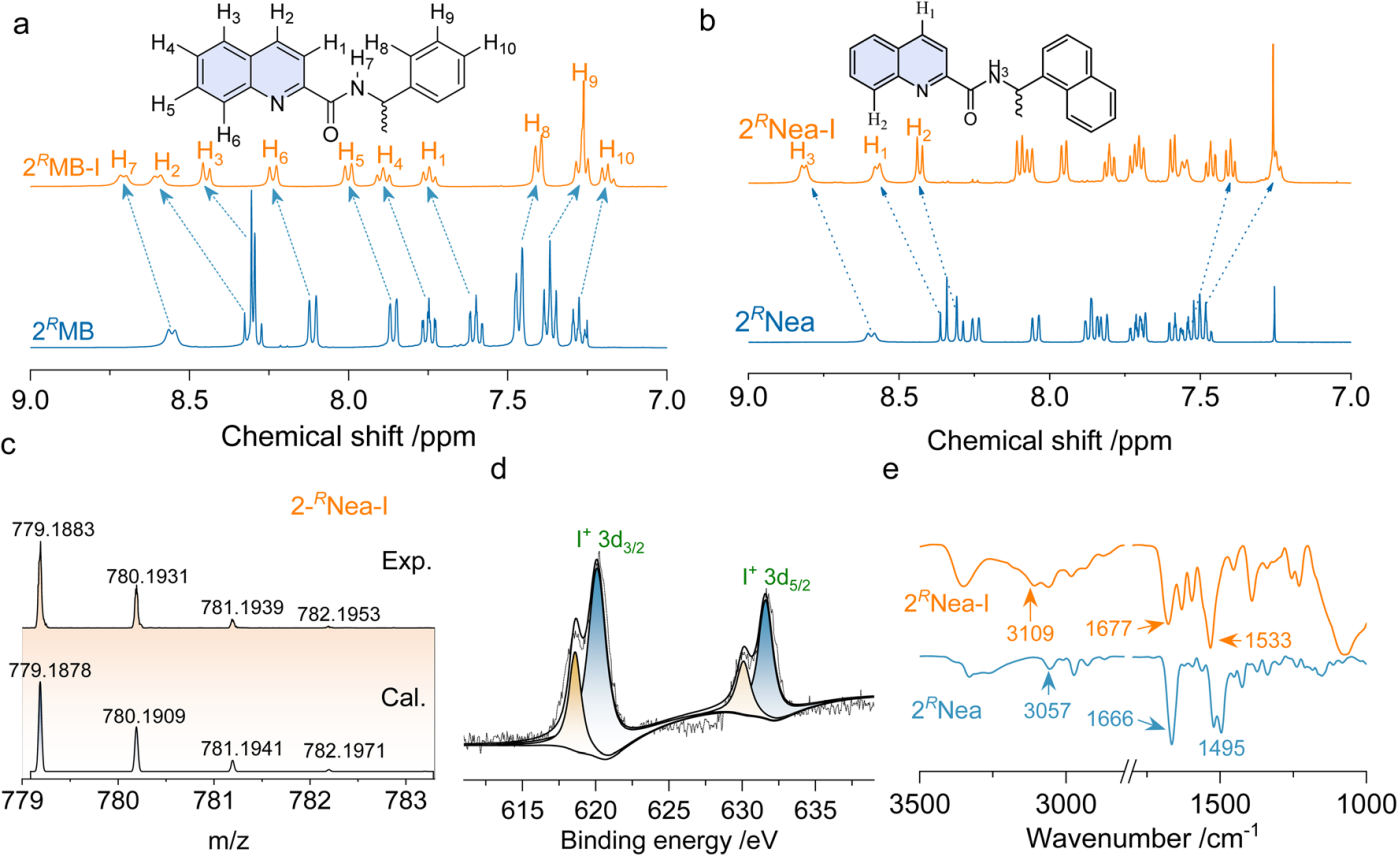
Characterizations of halogen bonded complexes. (a and b) ^1^H NMR spectra comparison of 2^*R*^MB/2^*R*^MB-I and 2^*R*^Nea/2^*R*^Nea-I respectively in CDCl_3_ (*c* = 10 mM, 298 K). (c) Calculated and experimental MS of 2^*R*^Nea-I. (d) XPS spectrum of 2^*R*^Nea-I. (e) Comparison of the FT-IR spectra of 2^*R*^Nea/2^*R*^Nea-I (KBr tablets).

Quinoline derivatives bearing chiral pendants at 2′ and 3′ positions were synthesized *via* amide condensation reactions. To synthesize halogen-bonding coupled supramolecular complexes, Ag(i) coordinated complexes were first synthesized, followed by the iodine substitution. The halogen-bonded products were fully characterized by ^1^H nuclear magnetic resonance (NMR), mass spectroscopy (MS), and other techniques. Fig. 1a represents the chemical shift variations after the halogen-bonded complexation of 2^*R*^MB. For the protons at the quinoline core, chemical shifts move to lower fields, while the chemical shifts on the chiral pendent exhibit high field shifts, and no new peaks are found. The p-holes on the cationic iodine are filled by the lone-pair electrons of the sp^2^ N, further diminishing the electron density of quinoline and enhancing the shielding effects.^42^ Consequently, the de-shielding effect is enhanced for the chiral pendent on the periphery. The chemical shift changes are also found in other analogs after the formation of the iodine complexes (Fig. 1b and S1†), without the observation of starting material residues or side products, implying that it was a quantitative reaction. In high-resolution MS spectra, the calculated values are consistent with the experimental results with well-defined isotopy resolution (Fig. 1c and S3†), indicating the formation of [N–I–N]^+^ complexes. X-ray photoelectron spectroscopy (XPS) that can detect the valency and types of elements were employed (Fig. 1d and S4†). Peaks at 620.1 eV and 631.6 eV are assigned as 3d_2/3_ and 3d_5/2_ band. The existence of I 3d spectrum indicates the generation of I^+^ species based on the previous reports.^43,44^ Fourier transform infrared spectroscopy (FT-IR) reflects the variations in bond information. In Fig. 1e, the peak at 3057 cm^−1^ is assigned to the stretching of protons on the quinoline cores, which shifted to 3109 cm^−1^ after complexation. Peaks at 1666 cm^−1^ and 1495 cm^−1^ are assigned to the vibrational stretching of C=O and C=C bonds in quinoline; all these peaks shifted to higher wavenumber regions.^45,46^ These shifts are contributed by the formation of iodine complexes that lower the electron density *via* halogen bonding. Through multiple characterizations, the successful synthesis of [N–I–N]^+^ complexes was confirmed.

**Fig. 2:**
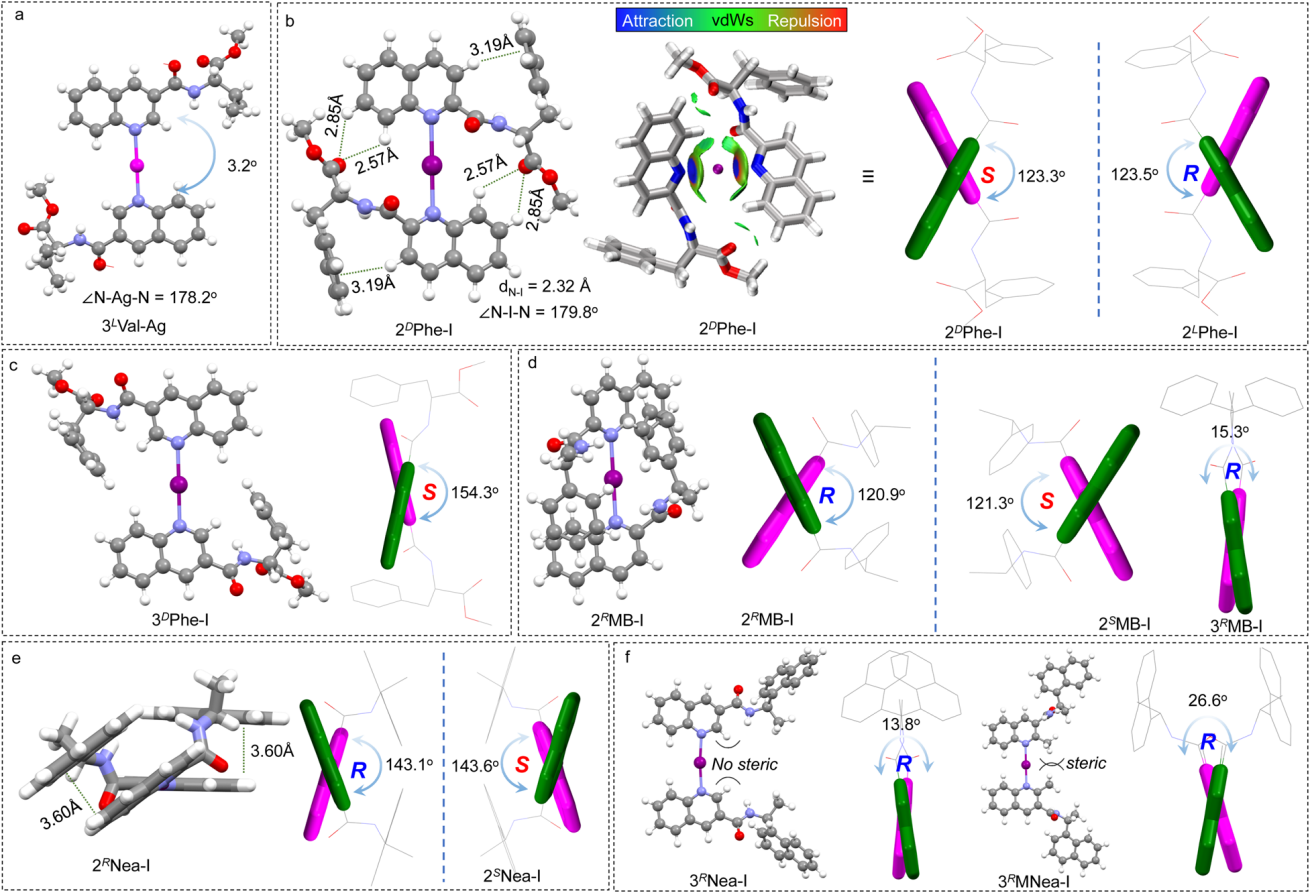
(a) X-ray crystal structure of the 3^L^Val-Ag complex. (b–f) DFT-optimized geometries of different halogen-bonded complexes. The *R* and *S* stand for the intrinsic axial chirality of biquinoline. Inset of (b) shows the IGMH analysis of 2^D^Phe-I (isosurface = 0.008 a.u.). For clarity, quinoline moieties are displayed in green and purple colors.

The X-ray crystal structure of the Ag(i)-coordinated intermediate is displayed in Fig. 2a. Expectedly, the N–Ag–N bond generated a bond angle of 178.2°, which linearly links the quinoline cores. This geometry provides a favorable precursor to fabricate a rotatable axial chiral complex. Quinoline segments pack in a *trans* mode (pendant on the opposite sides) that enables a slight screw sense with a dihedral angle of 3.2°. The dihedral screw adopts a preferred handedness that is determined by the chiral pendant, indicating that the absolute chirality of the pendant would determine the handedness of quinolines. This remote control may occur in both dilute solutions and solid states, and it arises from potential steric effects and adjacent packing. Due to the difficulties in obtaining iodo-compound crystals, density functional theory (DFT) calculations at the B3LYP-D3 def2TZVP level of theory with Grimme D3 dispersion correction (BJ-damping) were employed to unveil the geometries in dichloromethane using the SMD solvent model^47–49^ (Fig. 2b–f). 2^D^Phe-I complex with phenylalanine pendant features multiple intramolecular weak forces and internal folding (Fig. 2b). For instance, protons of quinoline with enhanced acidity form CH–π and H-bonds with benzene and carboxyl groups, respectively. The intramolecular folding was further verified by the independent gradient model based on the Hirshfeld partition (IGMH) analysis.^50^ The N–I distance was determined to be 2.32 Å, with an N–I–N angle of 179.8°, which is consistent with previous reports.^20,36^ The fine linearity of [N–I–N]^+^ halogen bond features rotatability and shape-resistance, preferably enabling the screw sense of quinolines. The dihedral angle was determined to be 123.3° (2^D^Phe-I), which was designated as a *S*-intrinsic aryl chirality. The screw sense shows an enantiomeric effect whereby 2^D^Phe-I gives opposite *R*-chirality with a similar dihedral angle (123.5°). Apparently, the steric effects of the chiral pendants are less prominent in the case of the 3′ position. For instance, 3^D^Phe-I gives a 154.3° dihedral angle, yet the complementary angle of 25.7° is relatively small considering the preferred *trans* conformation (Fig. 2c). The steric effects on the screw angle are also demonstrated in other examples (Fig. 2d–f). 2^*R*^MB-I shows a dihedral angle (120.9°) that is significantly larger than its 3′ counterpart (15.3°, 3^*R*^MB-I). The steric effect was further evidenced by a control experiment (Fig. 2f). The methyl group at the 2′ position of 3^*R*^Nea-I introduces considerable steric hindrance, due to which the dihedral angle increases from 13.8° to 26.6°. The results suggest that the chiral pendant on 2′ substitution favors the formation of axial chirality.

**Fig. 3:**
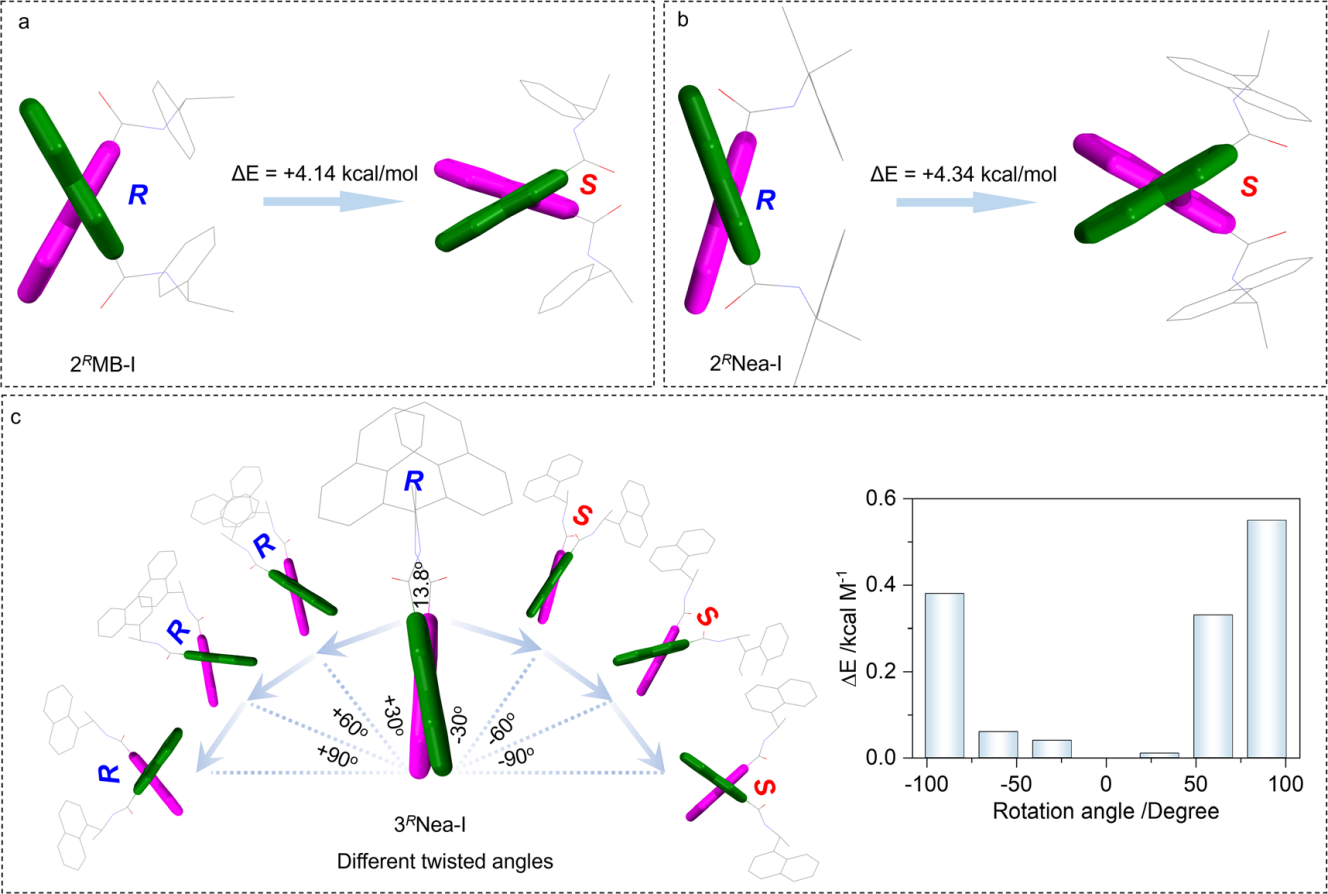
(a and b) Electronic energy gaps between 2^*R*^MB-I and 2^*R*^Nea-I adopting *R* or *S*-conformation, respectively. (c) Relative electronic energy variations with different dihedral angles of 3^*R*^Nea-I between the quinolines.

To elucidate the preferred chiral geometry with respect to system energy, examples of 2^*R*^MB-I and 2^*R*^Nea-I were compared (Fig. 3a and b). For 2^*R*^MB-I, the electronic energy of the *S*-isomer is energetically not stable compared to the *R*-isomer. An energy gap of 4.14 kcal mol^−1^ determines that 2^*R*^MB-I preferably adopted *R* screw sense. This energy difference is contributed by the steric effect of the chiral pendant, which hinders the racemization of biquinoline axial chirality. This energy difference could not be observed in the conventional axial compounds such as BINAP and biphenyl derivatives, whose *R*- and *S*-configuration share identical system energy and stability.^51^ In addition, the high steric effects and transformation barriers hinder the racemization of these chiral compounds. For the present case, increasing steric effects would slightly increase the selectivity (Fig. 3b). 2^*R*^Nea-I with a naphthalene moiety and larger steric effect gives a 4.34 kcal mol^−1^ energy difference, which is slightly larger than that of 2^*R*^MB-I. Compared to the 2′ substitutes, 3′ halogen bonded complexes show a very small energy gap between *R* and *S* (Fig. 3c), which is consistent with the DFT geometries and the small dihedral angles. Using 3^*R*^Nea-I, as the proof-of-concept, the impacts of rotation angles between quinolines on the energy were evaluated. The clockwise (*S* direction) and anti-clockwise (*R* direction) screw will gradually elevate the electronic energy gaps, which however are relatively small (less than 0.6 kcal mol^−1^). This energy difference suggests the poor selectivity towards the formation of homochiral axial complexes in solutions. The steric effect is a prominent factor to be considered in controlling axial chirality.

**Fig. 4:**
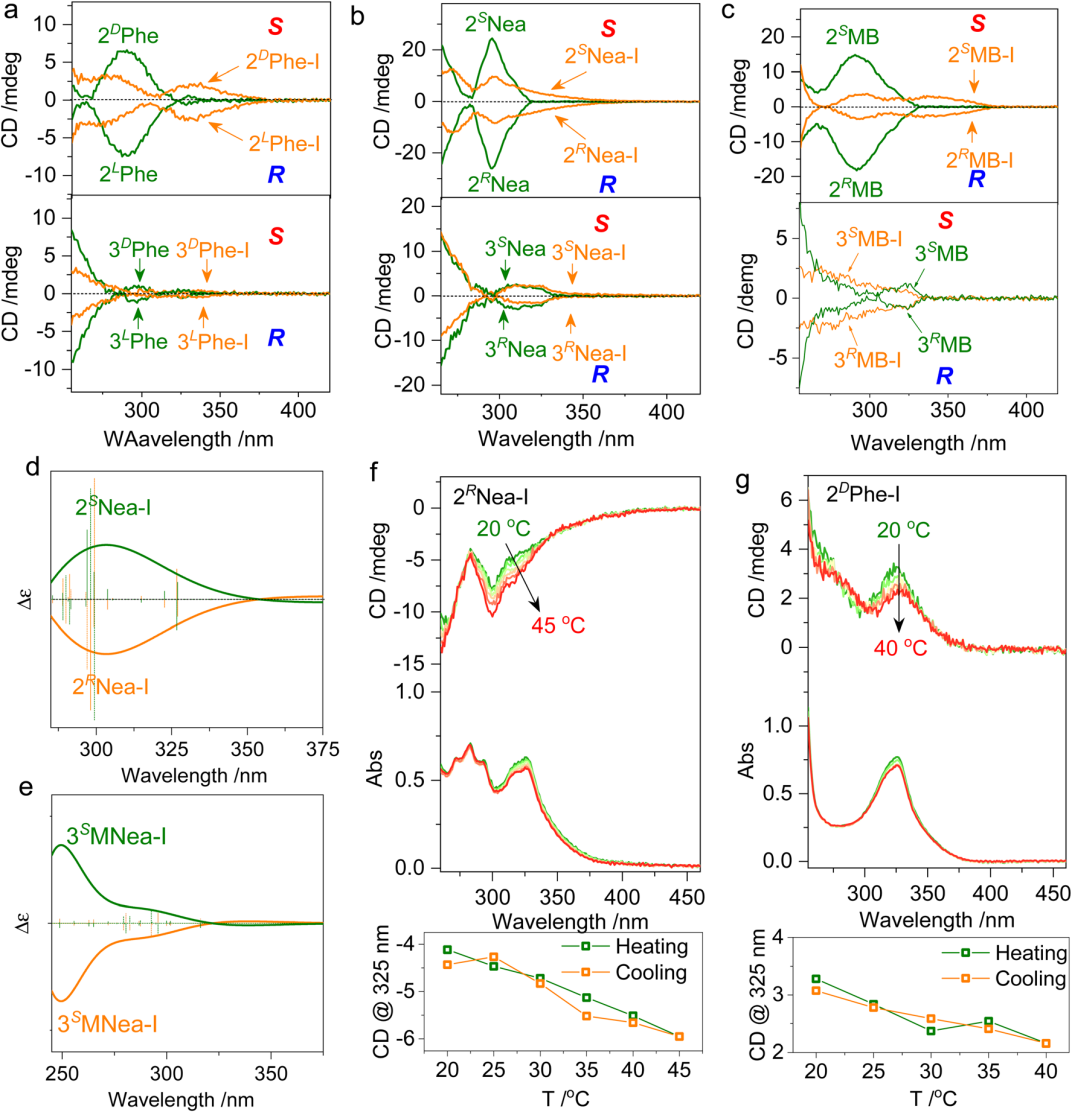
Chiroptical properties. (a–c) CD spectra of different starting compounds and halogen-bonded complexes in solutions (1 mM in CH_2_Cl_2_, path length = 1 mm, *R* and *S* stand for the observed handedness of halogen bonded complexes by DFT optimization). (d and e) Electronic CD calculation results of 2Nea-I and 3MNea-I respectively using B3LYP-D3 def2TZVP level of theory. Insets show the geometries of CD calculation. (f and g) Temperature-variable CD spectra of 2^*R*^Nea-I and 2^D^Phe-I (1 mM in CH_2_Cl_2_), respectively.

Chiroptical properties in solutions were evaluated (Fig. 4). The pristine starting compounds and the halogen-bonded complexes are highly soluble in many lab-used organic solvents without aggregation. The formation of ionic halogen bonding causes bathochromic shifts of absorption from 270 nm to around 330 nm (Fig. S5†). The absorption variation that occurs at quinoline segments initiates changes in the CD spectrum. In CH_2_Cl_2_ with a 1 mM concentration, 2^L^Phe-I and 2^D^Phe-I generate negative and positive Cotton effects over the absorption region (Fig. 4a). In contrast, the 3′ counterpart 3Phe-I is basically CD-mute without chiroptical activity, possibly caused by the co-presence of multiple chiral conformations and small steric effects, although 2′ and 3′ substitutes share the same chiral pendant. Active Cotton effects were also found in 2MB-I and 2Nea-I systems with enantiomeric selectivity (Fig. 4b and c). The signs of CD spectra are consistent with the designated *R* or *S* axial chirality as BINAP. An unpronounced steric effect is found in 3Nea-I, whose CD spectra barely change as compared to 3Nea. This indicates that the screw sense can easily undergo a transition between *R* or *S*, resulting in the failed formation of special axial chirality (Fig. 4b). However, after conjugating the methyl group as 3MNea-I with enhanced steric effects, an evolution in the Cotton effect was observed (Fig. S6†). In a word, the 3′ substitutes generate an externally racemic axially chiral complex due to unpronounced spatial steric effects, manifesting only monomeric point chirality signals in the solution. In contrast, the halogen-bonded complex of 2′ substitutes represents atropisomerism facilitated by hindered rotation; it achieves the preparation of supramolecular axially chiral motifs. The comparison of the CD spectra is in good agreement with the DFT-optimized geometries and designated handedness. Using the steric effect by region-selective substitution, axial chirality, and the corresponding chiroptical properties shall be readily controlled. The DFT-optimized structures of 2Nea-I and 3MNea-I were subjected to electronic CD calculations (Fig. 4d and e). 2^*S*^Nea-I and 2^*R*^Nea-I with *S*- and *R*-axial chirality show mirror Cotton effect curves. The sign of the CD spectra is in accordance with the experimental results, which implies they may adopt the proposed geometries in solutions. Then, the thermostability of the supramolecular chirality was evaluated (Fig. 4f, g and S7†). For example, by elevating 2^*R*^Nea-I solutions from 20 to 45 °C, the intensity of the Cotton effect was enhanced with slightly decreased absorbance. The variations are caused by the accelerated rotation as well as the de-solvation process, which is thermo-reversible. The cooling process witnesses the full recovery of Cotton effects with no hysteresis for both 2^*R*^Nea-I and 2^D^Phe-I. No racemization or decomposition was observed during heating–cooling cycles, indicating considerable thermo-resistance.

**Fig. 5:**
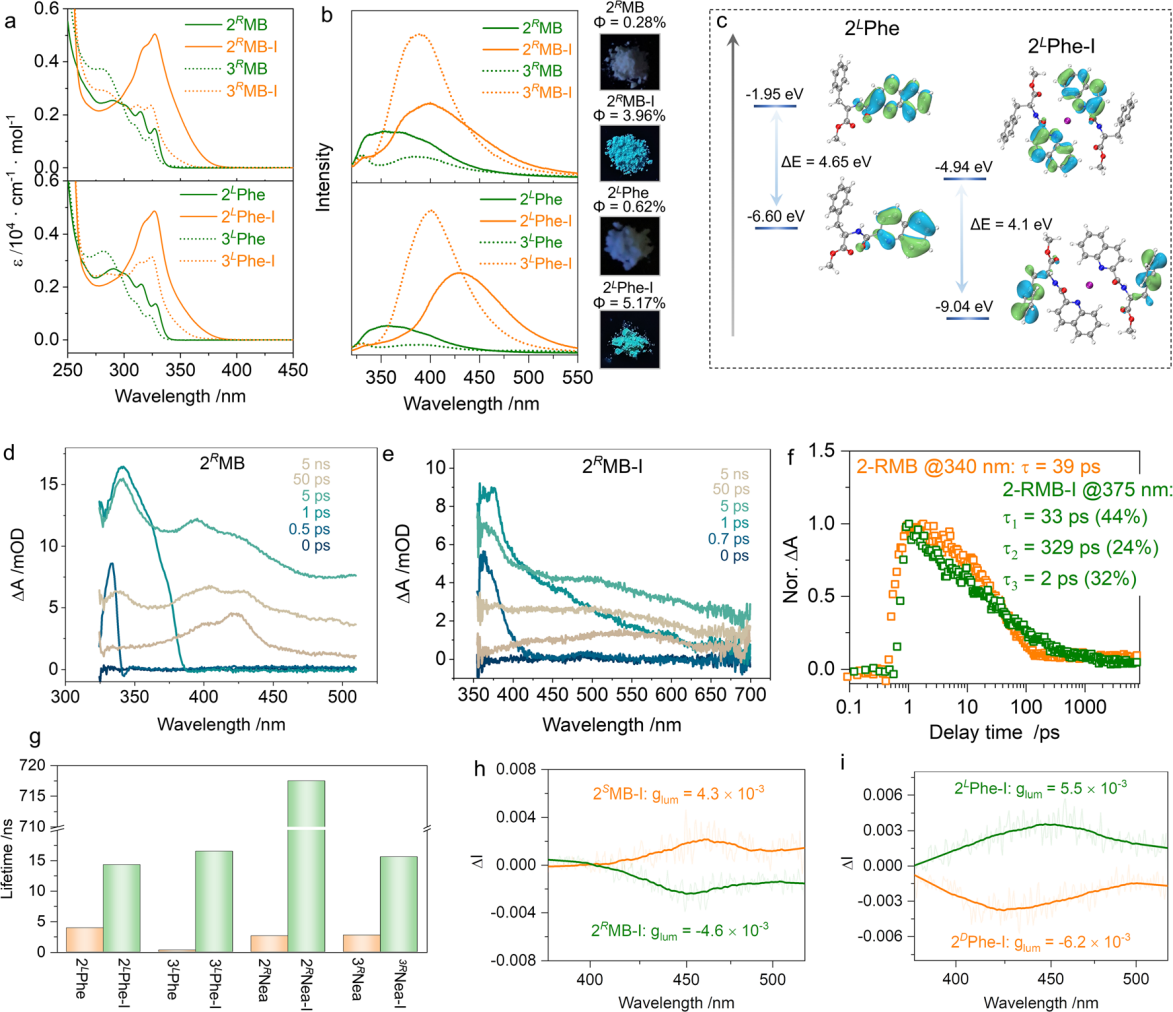
(a and b) Absorption and emission properties of 2^*R*^MB-I, 3^*R*^MB-I, 2^L^Phe-I, and 3^L^Phe-I, respectively (1 mM in CH_2_Cl_2_). Insets show the luminescent colors under UV light irradiation and quantum yields. (c) HOMO–LUMO orbitals and energy levels of 2^L^Phe-I. (d and e) The TA spectra of 2^*R*^MB and 2^*R*^MB-I at different delay times (10 mM in CH_2_Cl_2_). (f) The TA kinetic traces of 2^*R*^MB and 2^*R*^MB-I at specific probe wavelengths. (g) Lifetime comparison of different compounds measured in the solid state. (h and i) CPL spectra of 2MB-I and 2Phe-I measured in the PMMA film (*λ*_ex_ = 350 nm).

The formation of [N–I–N]^+^ ionic halogen bonding has dramatic impacts on the electronic ground and photoexcited states. In the absorption spectra (Fig. 5a), the multiple absorption bands of 2^*R*^MB with an extremum peak at 290 nm exhibited a bathochromic shift to 327 nm after the formation of halogen bonding. The absorption shifts were also observed in other supramolecular complexations with specific dependence on the substitution positions (Fig. S5†). The 2′ substitution possesses stronger absorption coefficients than those of the 3′ substitution even though the pristine compounds have similar absorption bands. This may be because the density of the electron cloud near the nitrogen atom in quinoline is reduced after the formation of [N–I–N]^+^ halogen bonds, which makes it easier for electron transitions to occur.^42,52^ Supramolecular complexation promotes a dramatic change in emission as well (Fig. 5b).^53,54^

2^*R*^MB-I and 2^L^Phe-I exhibit blue emission in CH_2_Cl_2_ with the maximum emission peak shifting from 350 nm for the monomers to 430 nm. Insets of Fig. 5b intuitively demonstrate the evolution of luminescence color in the powder state, whereby the emission in the visible region emerged. Meanwhile, the emission intensity of supramolecular complexes was enhanced, which may be caused by the formation of [N–I–N]^+^, inhibiting the non-radiative transition. The frontier orbitals of 2^L^Phe and 2^L^Phe-I are depicted in Fig. 5c. For 2^L^Phe, the highest occupied molecular orbital (HOMO) and lowest unoccupied molecular orbital (LUMO) are at −6.60 eV and −1.95 eV, respectively, with an energy gap of 4.65 eV; these are all located on the quinoline segments. In 2^L^Phe-I, the HOMO electron cloud is located on the phenyl moiety of the chiral pendant, while LUMO is located at the quinoline. The energy gap shrinks to 4.10 eV, corresponding to the bathochromic shifted absorption and emission wavelengths (Table S3†).^53^ We investigated the excited-state dynamics before and after the iodine complex formation using femtosecond transient absorption spectroscopy (TA, Fig. 5d–f and S8†). A 400 nm pump light was used for excitation, and the excited state absorption spectrum was collected in the wavelength range of 320 to 800 nm with a time window of 0–7.6 ns. In the ultrafast spectrum of 2^*R*^MB, there are three distinct excited state absorption (ESA) bands at 340 nm (ESA I), 400 nm (ESA II), and 420 nm (ESA III). Among them, ESA I decayed into a single exponential, and its lifetime was 39 ps. ESA II and ESA III did not decay completely within the time window tested. There were two obvious ESA signals in the ultrafast spectrum of 2^*R*^MB-I; the ESA I at 375 nm exhibited a triple-exponential decay with a decay lifetime of 94 ps, whereas ESA II at 550 nm did not completely decay in the whole-time window. It can be seen from the results that the lifetime of the excited state is extended to a large extent after the formation of the iodo-complex, resulting in slower radiative transition, which corresponds to the enlargement of the emission lifetime of the iodine complex (Fig. 5g). Chirality was transmitted to the photoexcited state, and CPL spectra were collected in the PMMA film to reduce any artifacts aroused by macroscopic anisotropy (Fig. 5h, i and S9†). Active CPL signals were observed at the maximum emission wavelength of ∼450 nm. 2^*R*^MB-I and 2^*S*^MB-I generated *s*- and *r*-CPL, respectively, with the same handedness as compared to 2^D^Phe-I and 2^L^Phe-I; this correlation is consistent with the CD signs shown in Fig. 4a and c. The luminescent asymmetry factors (*g*_lum_) reached ∼10^−3^, which is consistent with organic molecules reported in other works.^55,56^

**Fig. 6:**
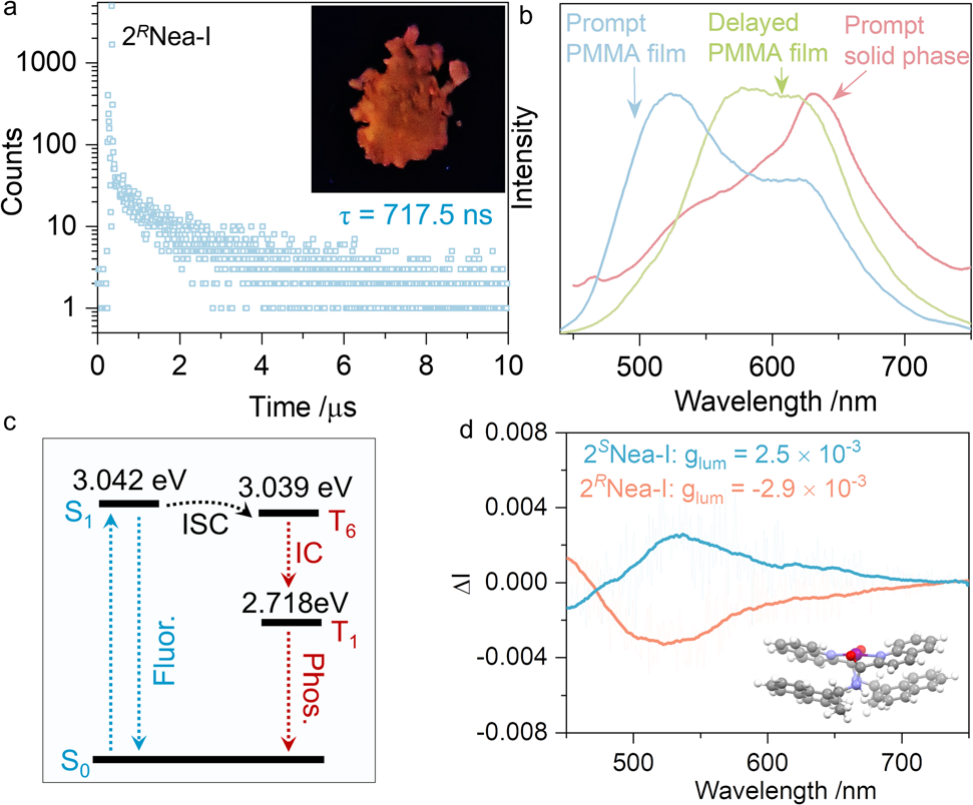
(a) Emission decay curve of 2^*R*^Nea-I in the solid state, as well as the red emission under UV light (delay time = 50 μs). (b) Comparison of the emission spectra of 2^*R*^Nea-I in different states. (c) The Jablonski diagram. (d) CPL of 2Nea-I measured in PMMA film. (*λ*_ex_ = 350 nm).

Unexpectedly, 2^*R*^Nea-I exhibited a longer lifetime as compared to other iodo-complexes (Fig. 5g). In the solid state, 2^*R*^Nea-I was red-emissive (Fig. 6a), while luminescent colors of other complexes remained blue or cyan. The lifetime of 717.5 ns can be assigned as a long-lived phosphorescence or thermally-activated delayed fluorescence. To validate the nature of such emissions, prompt and delayed emission spectra were collected in the polymethyl methacrylate (PMMA) thin films (Fig. 6b). The prompt emissions of the polymer matrix were located at ∼520 nm and 600 nm, respectively, while the delayed spectrum only had a peak at ∼600 nm, indicating that the red emission belongs to phosphorescence. In the solid state, even in the prompt state, the maximum peak at 630 nm was found due to the compact packing that hinders non-radiative emission and blocks the interference of oxygen. The Jablonski diagram is depicted in Fig. 6c. The first singlet excited state S_1_ with an energy level of 3.042 eV is close to the triplet excited state, T_6_ (3.039 eV), with a small Δ*E*_ST_ of 0.003 eV. After the intersystem crossing (ISC), the internal crossing (IC) occurs, finally giving rise to phosphorescence. The generation of phosphorescence benefits from the existence of iodine atoms with a heavy atom effect.^54,57,58^ However, the structural factors of the chiral pendant should be considered as well, due to 2Nea-I exclusively demonstrating the long-lived emission. A particular structural feature of 2Nea-I is the formation of intramolecular π–π stacking between quinoline and adjacent naphthalene moieties (3.60 Å),^59^ which does not exist in 3Nea-I or other complexes (Fig. 2e). This feature would rigidly anchor the conformation that diminishes the nonradiative emission to facilitate the ISC process. In addition to the above results, circularly polarized phosphorescence (CPP, Fig. 6d) was realized with *g*_lum_ of ∼10^−3^, which is consistent with other CPP materials.^60,61^

## Conclusions

In summary, we successfully employed a [N–I–N]^+^-type halogen bond as a general protocol to construct supramolecular axial chiral compounds. Quinoline derivatives with chiral pendants at the 2′ and 3′ positions, coupled by halogen bonds, generated supramolecular dimers that exhibited axial chiral features as indicated by experiments and DFT calculations. The supramolecular complexes and chirality showed prominent impacts on steric hindrance, which influenced the chirality transfer from the pendant to the aryl cores. The strong binding energy of [N–I–N]^+^-type halogen bond allows resistance to solvation and thermal treatment. Luminescent evolution was observed to be triggered by halogen bonds, inducing multiple emissions and chiroptical properties, whereby the CPL and CPP were realized. This work established a new strategy to build axial supramolecular complexes, paving an avenue for supramolecular asymmetric catalysis and optics.

## Experimental

Materials, experimental details, and additional CD, ^1^H NMR, ^13^C NMR, and MS spectra can be found in the ESI.†

## Data availability

Additional experimental data supporting this article are included in the ESI.† Reasonable requests for additional information can be made to the corresponding authors.

## Author contributions

S. An carried out the main experiments and data analysis. P. Xing and A. Hao proposed the assumption and wrote the paper.

## Conflicts of interest

There are no conflicts to declare.

## Supplementary Material

SC-014-D3SC03170E-s001

SC-014-D3SC03170E-s002
