# Supplementary material for: Supramolecular axial chirality in [N–I–N]+-type halogen bonded dimers
Source: Chem Sci. 2023 Aug 29;14(37):10194–202. doi: 10.1039/d3sc03170e (PMC10530288; doi:10.1039/d3sc03170e)
Supplement: SC-014-D3SC03170E-s001 [file SC-014-D3SC03170E-s001.pdf]

Electronic Supplementary Material (ESI) for Chemical Science.
This journal is © The Royal Society of Chemistry 2023

Electronic Supplementary Information

for

Supramolecular Axial Chirality in $[N\text{-}I\text{-}N]^+$ Type Halogen Bonded Dimers

Shuguo An,[†] Aiyou Hao[†] and Pengyao Xing*,[†]

[†]Key Laboratory of Colloid and Interface Chemistry of Ministry of Education and School of Chemistry and Chemical Engineering, Shandong University, Jinan 250100, People's Republic of China. Email: xingpengyao@sdu.edu.cn

Contents

1. General information 1
  1.1 Materials 1
  1.2 Characterizations 2
  1.3 Synthesis 3
2. Additional experimental datas 21
3. Optimized geometries 29
Reference 43

1. General information

1.1 Materials

Silver tetrafluoroborate ($AgBF_4$) was purchased from Energy Chemical Co., Ltd (China). Quinoline-3-carboxylic acid and quinoline-2-carboxylic acid were purchased from Bide Pharmatech Ltd. (S)-1-phenylethan-1-amine, (R)-1-phenylethan-1-amine, (S)-1-(naphthalen-1-yl)ethan-1-amine, (R)-1-(naphthalen-1-yl)ethan-1-amine were purchased from Shanghai Bide Medical Technology Co., LTD. Polymethyl methacrylate (PMMA, *Mw* = 350, 000 was purchased from Shanghai Macklin Biochemical Co., Ltd (China). *L* and *D*-Phenylalanine methyl ester hydrochloride (*L*

and *D*-Phe-OMe.HCl), N-(3-Dimethylaminopropyl)-N'-ethylcarbodiimide hydrochloride (EDC), 1-Hydroxybenzotriazole (HOBT), 4-Dimethylaminopyridine (DMAP) were purchased from Heowns Biochem LLC. Other reagents and solvents were purchased from Guoyao Chemical Reagent Co. Ltd, Shanghai. All chemicals were used without further purification.

1.2 Characterizations

Nuclear magnetic resonance (NMR) spectra were obtained using Bruker AM-400 spectrometer at room temperature with tetramethylsilane (TMS) as the reference. High resolution mass (HRMS) spectra were collected with an Ultra-performance liquid chromatography coupled to quadrupole Tine-of-flight spectrometer (Bruker, impact II), the charging voltage and dry heater temperature were set at 2000 V and 180 ^oC respectively. Phosphorescence quantum yield and life time were recorded *via* FLS-1000 photoluminescence spectrometer from Edinburgh Instruments Ltd. (UK). Circular dichroism (CD) and circularly polarized luminescence (CPL) spectra were measured with an Applied Photophysics Chirascan V100 model (UK). X-*ray* photoelectron spectroscopy (XPS) measurements were performed on a Thermo Fisher-VG Scientific (ESCALAB 250Xi) photoelectron spectrometer equipped with a monochromatic Al Kα X-*ray* source (*hv* = 1486.8 eV). The sample was loaded into a custom built air-free sample holder, under a N_2 atmosphere. Prior to data collection, a baseline vacuum of 1.07×10^{-9} mbar was achieved. For high resolution scans, a band pass energy of 30 eV was used. Binding energies were calibrated using the C1s peak of adventitious carbon at 284.8 eV. Transient absorption spectra were collected by Femtosecond Transient Absorption Spectrometer (Ultrafast, USA). An Astrella Ti: Sapphire laser system from Coherent (USA) was used as a light source, which operates at a 1 kHz repetition rate, generating 70-fs pulses at 800 nm. The ~70 fs pump laser pulse was generated by a regenerative amplifier system and the optical parametric amplifier (Coherent, Solo). A small portion of the laser fundamental was focused into a sapphire plate to produce a supercontinuum in the visible range, which overlapped with the pump in time and space. Transient spectra and kinetic curves were recorded after removing spikes, subtracting background, and applying chirp corrections using software supplied by

Ultrafast Systems. Single-crystal X-ray diffraction data was collected on a Rigaku Oxford Diffraction XtaLAB Synergy diffractometer equipped with a HyPix-6000HE area detector (Japan) at 173 K using Cu Kα (λ = 1.54184 Å) from PhotonJet micro-focus X-ray Source. The diffraction images were processed and scaled using the CrysAlisPro software. These structures were solved using the charge-flipping algorithm, as implemented in the program SUPERFLIP 2 and refined by full-matrix least-squares techniques against *Fo* 2 using the SHELXL program3 through the OLEX2 interface.4 Hydrogen atoms at carbon were placed in calculated positions and refined isotropically by using a riding model.

1.3 Synthesis

Synthesis of quinoline derivatives

All quinoline derivatives are obtained using a similar method, with the synthesis of 2^{R}NEA serving as an example. 865 mg (5 mmol) of quinoline-2-carboxylic acid and 1.04 g (6 mmol, 1.2eq) of (R)-1-(naphthalen-1-yl)ethan-1-amine are added to 100 mL of *N,N*-Dimethylformamide (DMF) along with 200 mg of DMAP, 200 mg of HOBT, and 2 g of EDC, and the mixture was stirred at room temperature for 24 hours. After completion of the reaction, the organic phase was extracted 5 times with dichloromethane and water, and the collected organic phase is dried with anhydrous sodium sulfate. After removal of the solvent under vacuum, the product 2^{R}Nea is further purified by column chromatography. For 2^{L}Phe and 2^{D}Phe, 1 mL of triethylamine should be added during synthesis to neutralize the hydrochloric acid from *L* and *D*-Phenylalanine methyl ester hydrochloride.

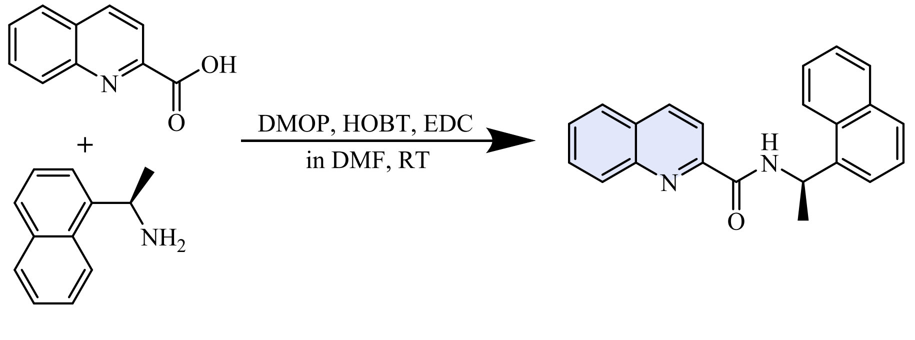

Scheme S1 Synthesis route of quinoline derivatives.

Synthesis of I-complex

The synthesis route of I-complexes is depicted in **Scheme S2**, and the details as follows.[S1,S2] 0.1 mmol of quinoline derivative was added into a 25 mL round-bottom flask with 5 mL of dichloromethane. 19.4 mg of $AgBF_4$ was dissolved in 1 mL of methanol in a flask. The reaction was allowed to proceed for one hour until complete. 30.5 mg of iodine was dissolved in dichloromethane, which was added drop-wisely to the flask (a white precipitate will immediately form), and the reaction was further conducted for another hour. The solvents were removed under vacuum. The residue was dissolved in a small amount of dichloromethane and n-hexane was added to induce crystallization. The supernatant was removed by centrifuge. The process was repeated several times until the supernatant turned into colorless. The resulting solid was vacuum-dried, which was stored in a sealed container. (**Notes**: some samples are sensitive to moisture, and the required samples for testing should be prepared and measured as soon as possible.)

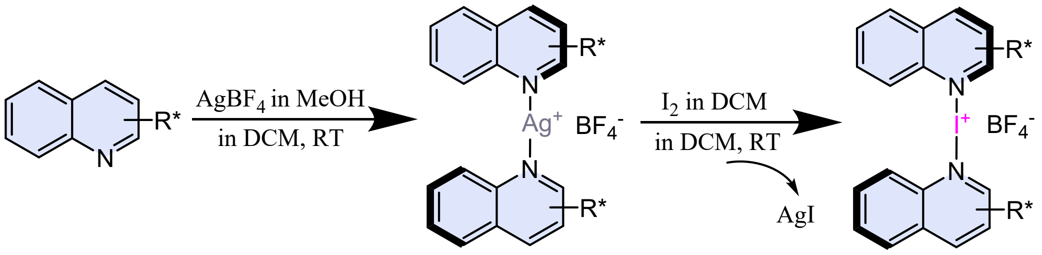

Scheme S2. Synthesis route of I-complexes.

1.4 ^{1}H and ^{13}C NMR and HRMS spectra of synthesized compounds

2*ᴰ*Phe

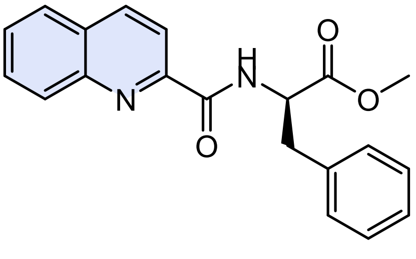

Purified by column chromatography on a silica gel column using dichloromethane and petroleum ether as the eluent to give the target compound as a white solid (1.30 g, 78% yield). ^{1}H NMR (400 MHz, Chloroform-*d*) δ 8.67 (d, *J* = 8.3 Hz, 1H), 8.27 – 8.15 (m, 2H), 8.05 (dd, *J* = 8.6, 1.2 Hz, 1H), 7.80 (dd, *J* = 8.2, 1.4 Hz, 1H), 7.69 (ddd, *J* = 8.4,

6.9, 1.4 Hz, 1H), 7.55 (ddd, J = 8.1, 6.9, 1.2 Hz, 1H), 7.23 – 7.14 (m, 5H), 5.05 (dt, J = 8.4, 6.1 Hz, 1H), 3.68 (s, 3H), 3.23 (d, J = 6.1 Hz, 2H). ^{13}C NMR (101 MHz, Chloroform-*d*) δ 171.90, 164.06, 149.00, 146.40, 137.62, 136.06, 130.19, 129.88, 129.43, 129.39, 128.64, 128.11, 127.70, 127.16, 118.83, 53.58, 52.39, 38.35. HRMS (TOF) m/z [M+H]$^+$: calcd for $C_{20}H_{19}N_2O_3^+$ at 335.1396, found at 335.1404.

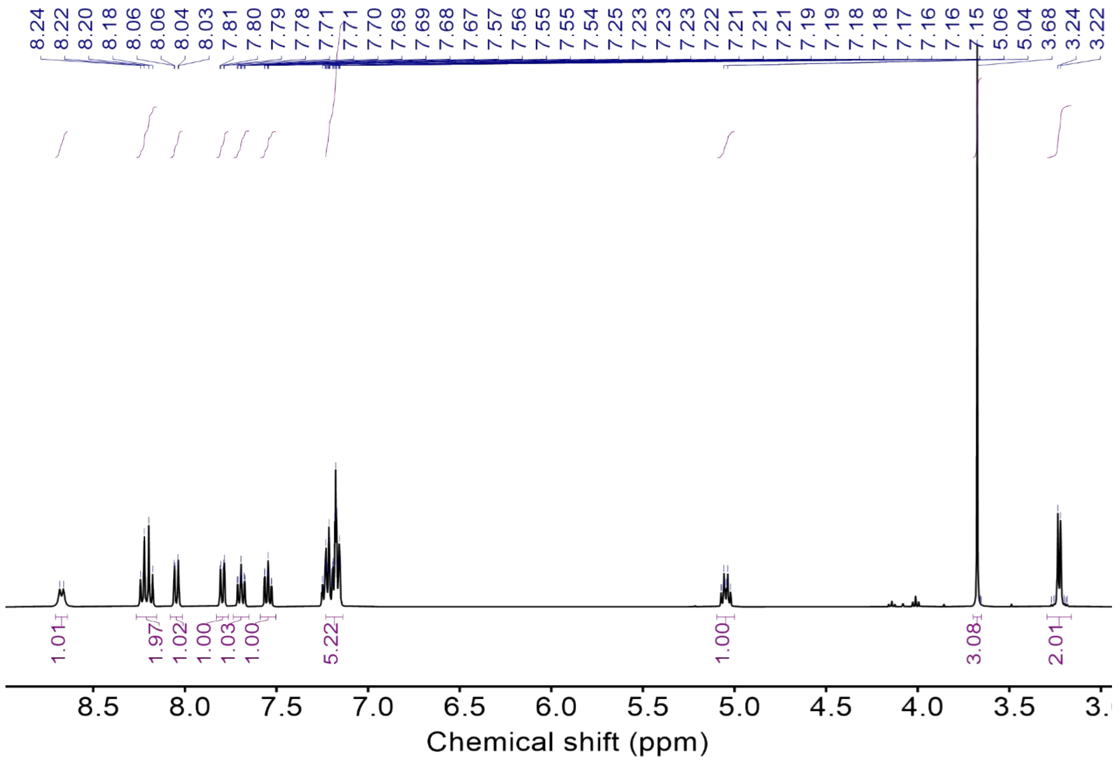

^{1}H NMR spectrum of **2^{L}Phe** ($CDCl_3$, 400 MHz, 298 K).

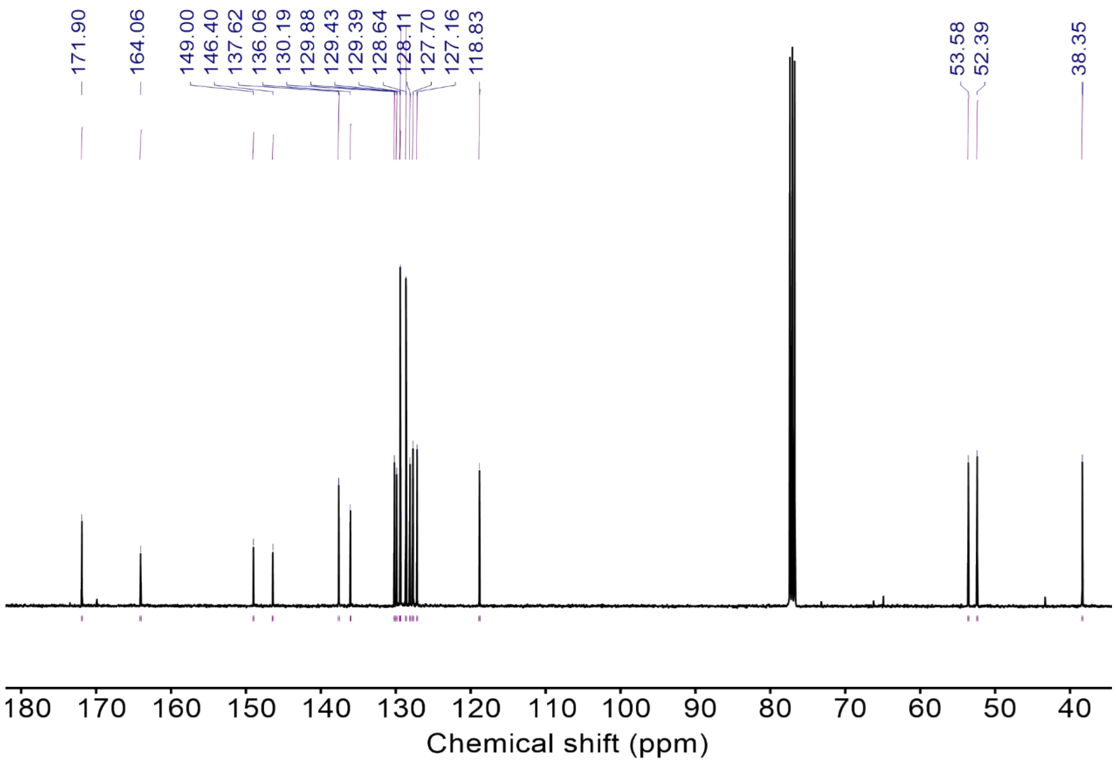

^{13}C NMR spectrum of **2^{L}Phe** ($CDCl_3$, 101 MHz, 298 K).

2^{L}Phe-I

25% yield, white solid. ^{1}H NMR (400 MHz, Chloroform-*d*) δ 8.71 (d, *J* = 8.2 Hz, 1H), 8.62 (d, *J* = 7.8 Hz, 1H), 8.30 (dd, *J* = 13.0, 8.7 Hz, 2H), 7.99 (d, *J* = 8.2 Hz, 1H), 7.92 (t, *J* = 7.8 Hz, 1H), 7.75 (t, *J* = 7.6 Hz, 1H), 7.28 – 7.19 (m, 5H), 5.11 – 5.01 (m, 1H), 3.73 (s, 3H), 3.30 (qd, *J* = 14.0, 6.7 Hz, 2H).

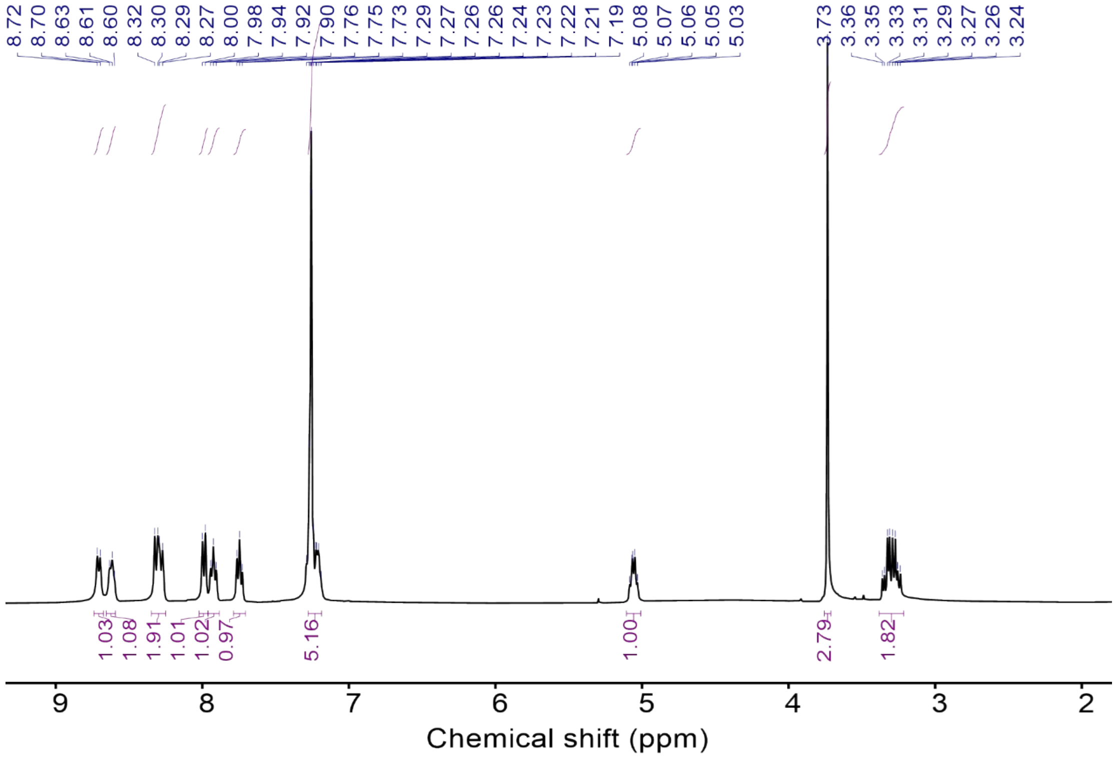

^{1}H NMR spectrum of **2^{L}Phe-I** ($CDCl_3$, 400 MHz, 298 K).

2^{R}MB

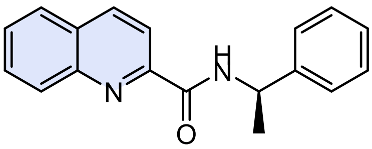

Purified by column chromatography on a silica gel column using dichloromethane and petroleum ether as the eluent to give the target compound as a colorless viscous liquid. After being left for several days, colorless crystals will form (966 mg, 70% yield). ^{1}H NMR (400 MHz, Chloroform-*d*) δ 8.59 (d, *J* = 8.4 Hz, 1H), 8.38 – 8.28 (m, 2H), 8.15 (dd, *J* = 8.5, 1.1 Hz, 1H), 7.89 (dd, *J* = 8.2, 1.5 Hz, 1H), 7.78 (ddd, *J* = 8.5, 6.9, 1.5 Hz, 1H), 7.63 (ddd, *J* = 8.2, 6.9, 1.2 Hz, 1H), 7.53 – 7.46 (m, 2H), 7.45 – 7.36 (m, 2H), 7.35 – 7.27 (m, 1H), 5.49 – 5.37 (m, 1H), 1.72 (d, *J* = 6.9 Hz, 3H). ^{13}C NMR (101 MHz, Chloroform-*d*) δ 163.64, 149.80, 146.49, 143.35, 137.50, 130.08, 129.73, 129.35, 128.73, 127.90, 127.77, 127.37, 126.33, 118.94, 48.96, 22.10. HRMS (TOF) m/z $[M+H]^+$: calcd for $C_{18}H_{17}N_2O^+$ at 277.1341, found at 277.1263.

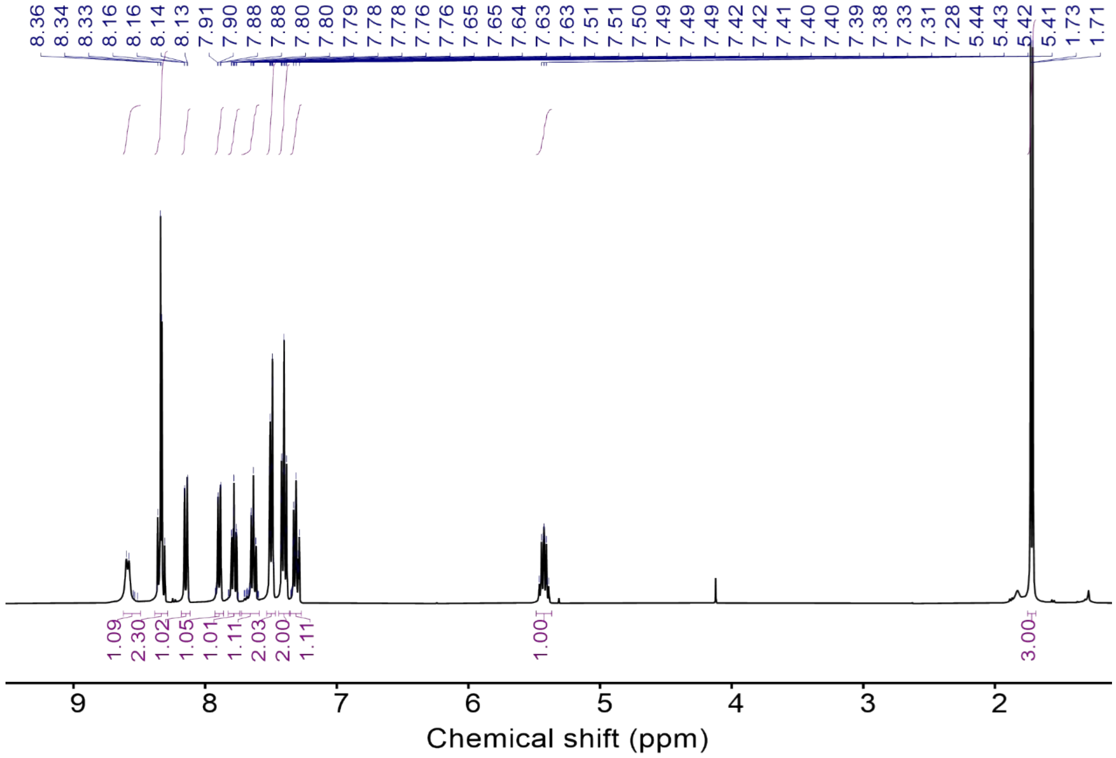

^{1}H NMR spectrum of **2^{R}MB** ($CDCl_3$, 400 MHz, 298 K).

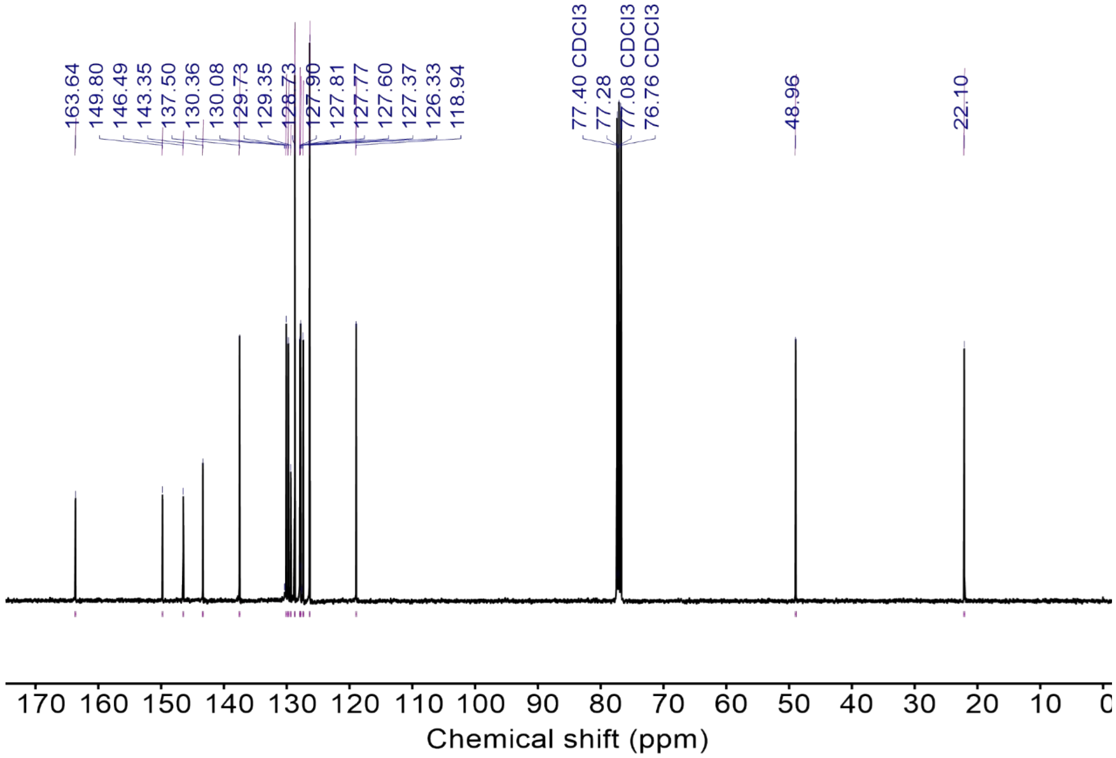

^{13}C NMR spectrum of **2^{R}MB** ($CDCl_3$, 101 MHz, 298 K).

2^{R}MB-I

35% yield, white solid. ^{1}H NMR (400 MHz, Chloroform-*d*) δ 8.73 (d, *J* = 7.8 Hz, 1H), 8.62 (d, *J* = 8.4 Hz, 1H), 8.47 (d, *J* = 8.5 Hz, 1H), 8.26 (d, *J* = 8.5 Hz, 1H), 8.02 (d, *J* = 8.2 Hz, 1H), 7.91 (t, *J* = 7.8 Hz, 1H), 7.77 (t, *J* = 7.5 Hz, 1H), 7.46 – 7.39 (m, 2H), 7.29 (t, *J* = 7.5 Hz, 2H), 7.21 (t, *J* = 7.3 Hz, 1H), 5.31 (p, *J* = 7.2 Hz, 1H), 1.70 (d, *J* = 7.0 Hz, 3H).

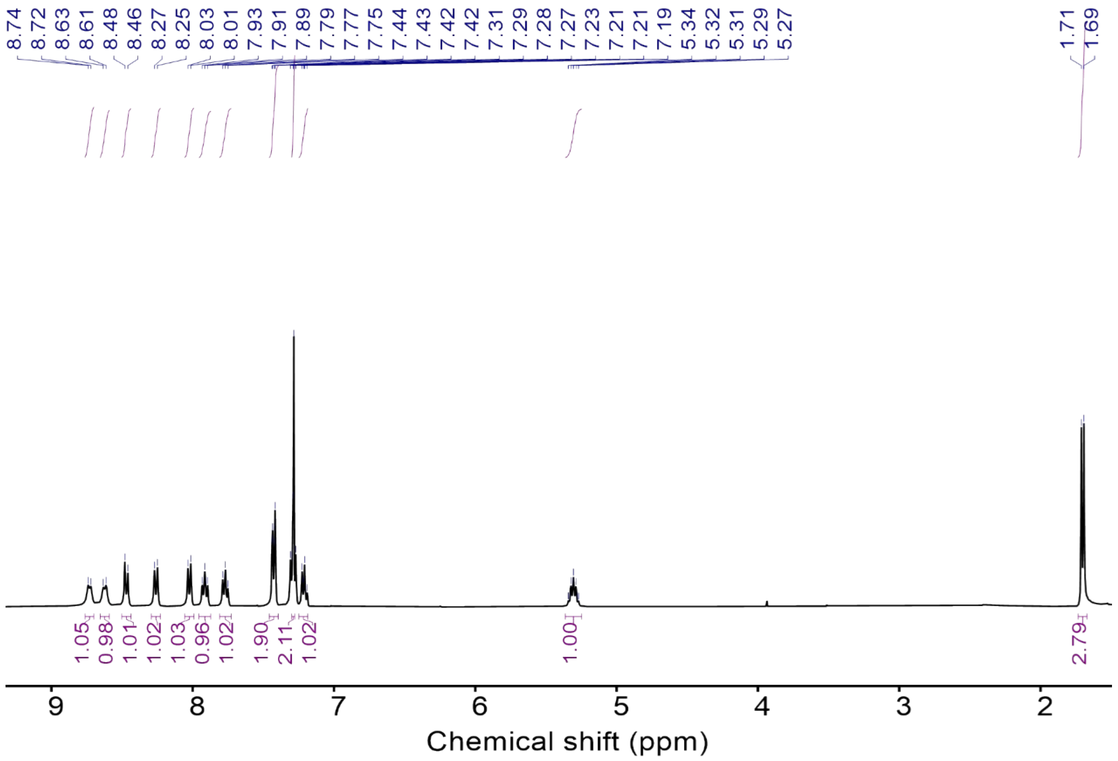

^{1}H NMR spectrum of **2^{R}MB-I** ($CDCl_3$, 400 MHz, 298 K).

2^{R}Nea

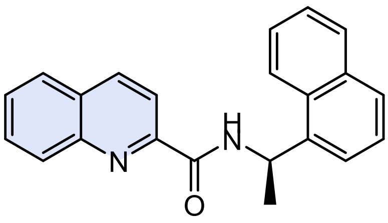

Purified by column chromatography on a silica gel column using dichloromethane and petroleum ether as the eluent to give the compound as white powder (1.53 mg, 94% yield). ^{1}H NMR (400 MHz, Chloroform-*d*) δ 8.59 (d, *J* = 8.7 Hz, 1H), 8.35 (d, *J* = 8.5 Hz, 1H), 8.30 (dd, *J* = 8.5, 0.8 Hz, 1H), 8.27 – 8.21 (m, 1H), 8.05 (dq, *J* = 8.5, 0.9 Hz,

1H), 7.86 (td, J = 7.9, 1.7 Hz, 2H), 7.82 (dt, J = 8.3, 1.1 Hz, 1H), 7.76 – 7.64 (m, 2H), 7.58 (ddd, J = 8.1, 6.9, 1.2 Hz, 1H), 7.58 – 7.44 (m, 3H), 6.20 (dq, J = 8.7, 6.9 Hz, 1H), 1.85 (d, J = 6.8 Hz, 3H). ^{13}C NMR (101 MHz, Chloroform-*d*) δ 163.42, 149.67, 146.44, 138.58, 137.51, 133.98, 131.22, 130.05, 129.68, 129.35, 128.80, 128.33, 127.89, 127.73, 126.56, 125.82, 125.34, 123.49, 122.72, 118.98, 44.94, 21.30. HRMS (TOF) m/z $[M+H]^+$: calcd for $C_{22}H_{19}N_2O^+$ at 327.1497, found at 327.1490.

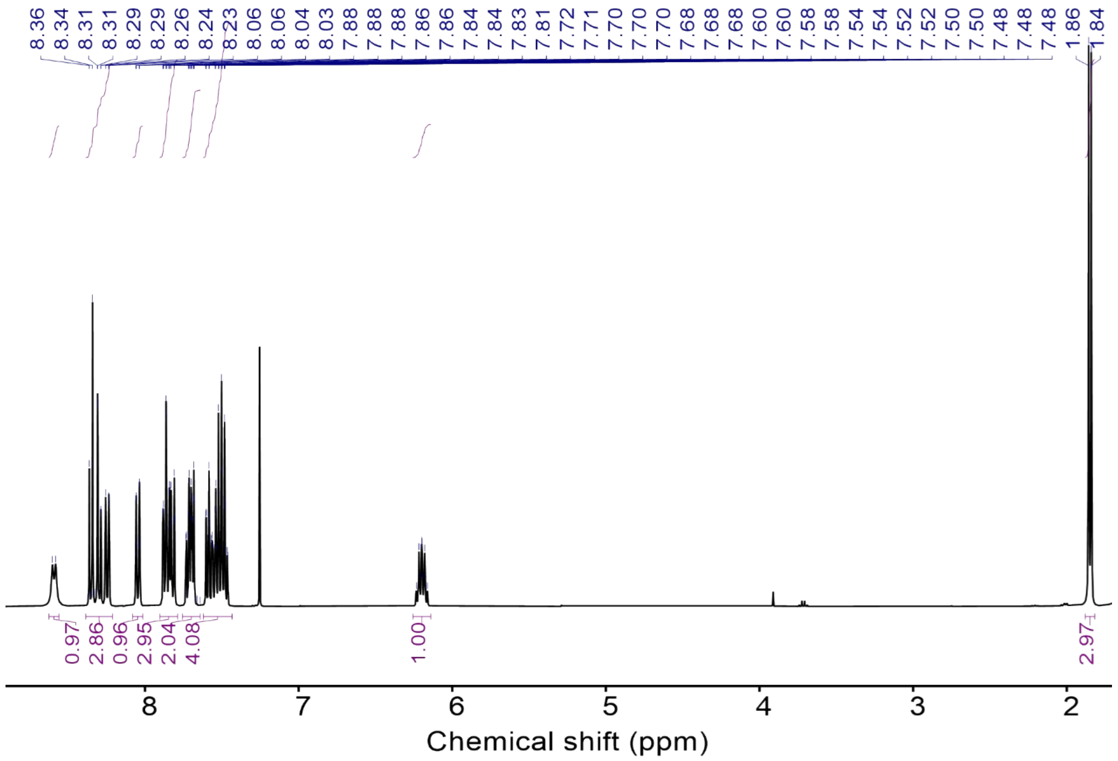

^{1}H NMR spectrum of **2^{R}Nea** ($CDCl_3$, 400 MHz, 298 K).

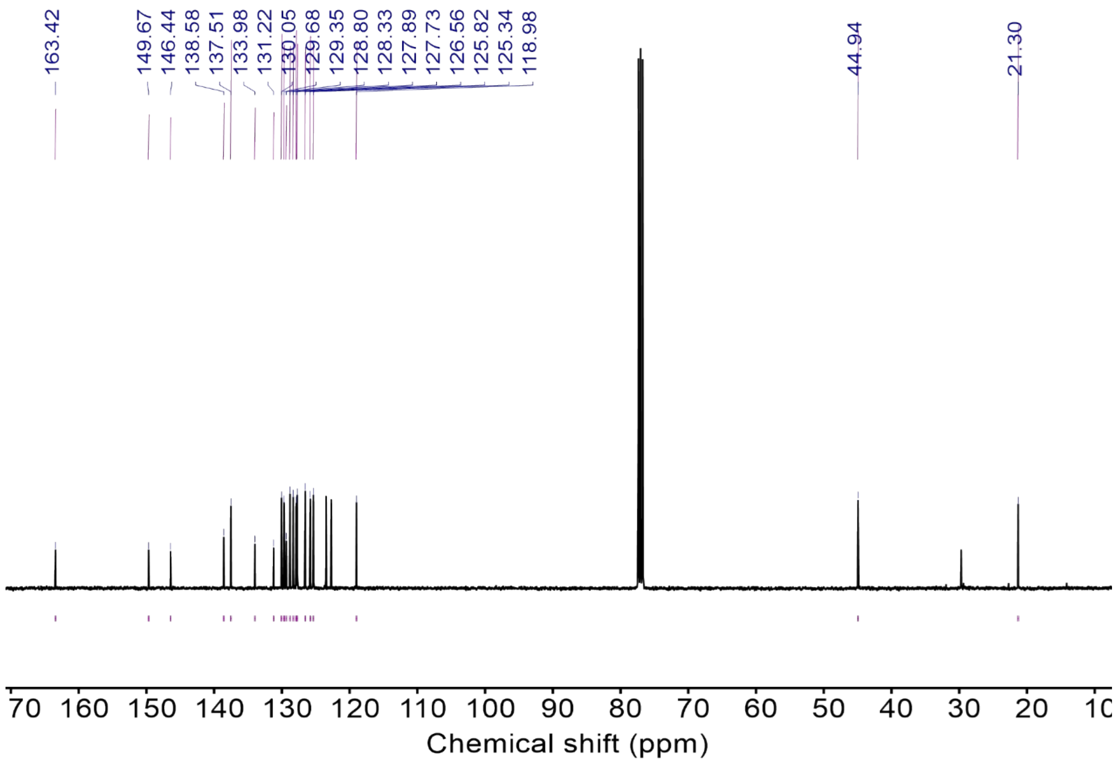

^{13}C NMR spectrum of **2*^R^*Nea** ($CDCl_3$, 101 MHz, 298 K)

2^{R}Nea-I

35% yield, yellow powder.^1H NMR (400 MHz, Chloroform-*d*) δ 8.91 (s, 1H), 8.65 (s, 1H), 8.47 (s, 1H), 8.17 (d, *J* = 8.2 Hz, 1H), 8.07 (d, *J* = 8.4 Hz, 1H), 7.99 (d, *J* = 8.0 Hz, 1H), 7.84 (d, *J* = 8.2 Hz, 1H), 7.76 (t, *J* = 7.0 Hz, 1H), 7.70 (d, *J* = 7.8 Hz, 1H), 7.61 (s, 1H), 7.56 – 7.45 (m, 2H), 7.41 (t, *J* = 7.2 Hz, 1H), 7.23 (s, 1H), 5.94 (s, 1H), 1.78 (d, *J* = 6.2 Hz, 3H).

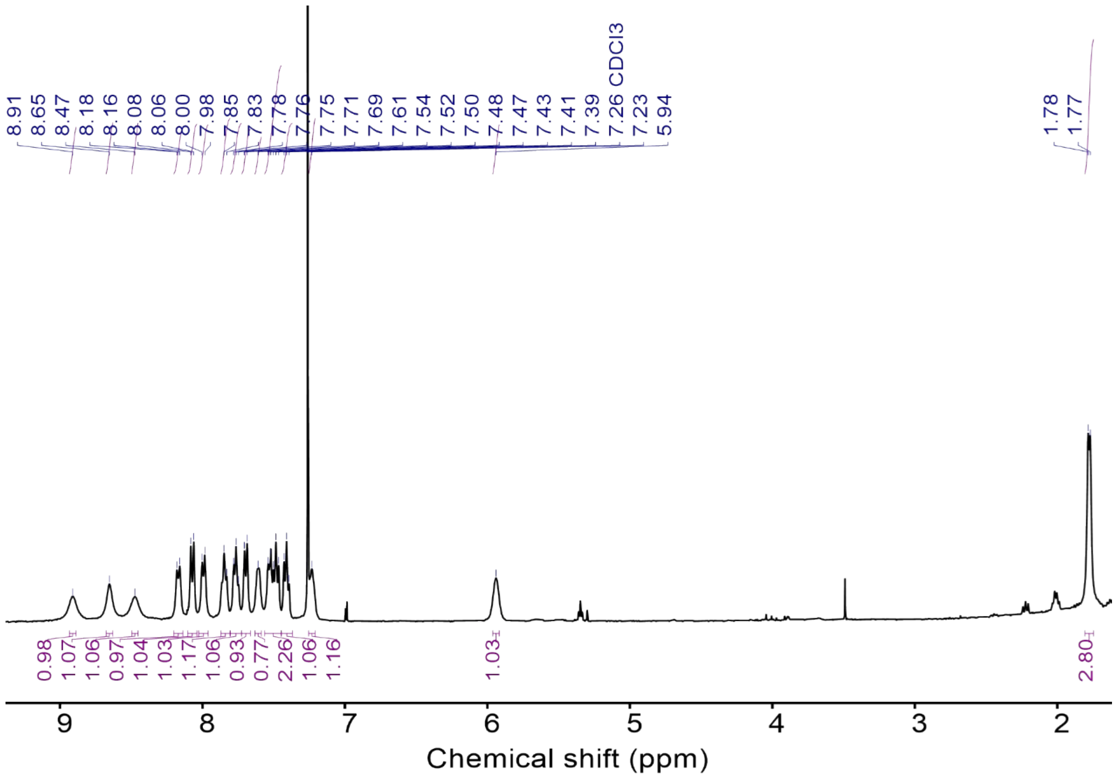

^{1}H NMR spectrum of **2^{R}Nea-I** (CDCl$_3$, 400 MHz, 298 K).

3^{R}MB

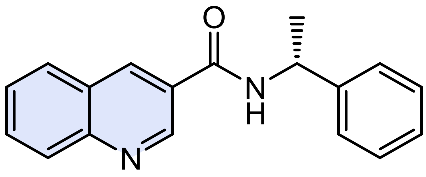

Purified by column chromatography on a silica gel column using dichloromethane and petroleum ether as the eluent to give the compound as a pale-yellow solid (828 mg, 60% yield). ^{1}H NMR (400 MHz, Chloroform-*d*) δ 9.24 (d, *J* = 2.3 Hz, 1H), 8.53 (d, *J* = 2.3 Hz, 1H), 8.07 (dd, *J* = 8.4, 1.1 Hz, 1H), 7.85 – 7.70 (m, 2H), 7.55 (ddd, *J* = 8.1, 6.8, 1.2 Hz, 1H), 7.46 – 7.21 (m, 5H), 7.01 (d, *J* = 7.8 Hz, 1H), 5.38 (p, *J* = 7.1 Hz, 1H), 1.63 (d, *J* = 6.9 Hz, 3H). ^{13}C NMR (101 MHz, Chloroform-*d*) δ 164.89, 149.02, 148.24, 142.85, 135.68, 131.23, 129.17, 128.79, 128.70, 127.61, 127.48, 127.10, 126.85, 126.35, 49.58, 21.65. HRMS (TOF) m/z [M+H]$^{+}$: calcd for $C_{18}H_{17}N_2O^{+}$ at 277.1341, found at 277.1326.

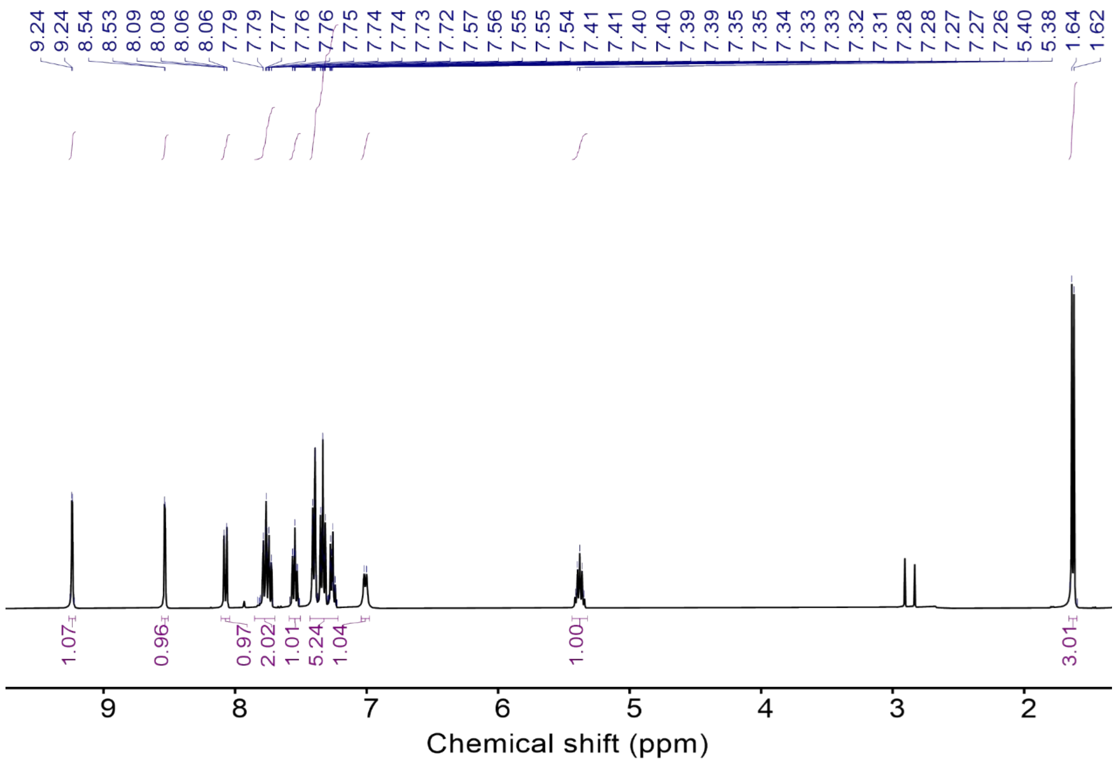

^{1}H NMR spectrum of $\mathbf{3^{\mathit{R}}MB}$ ($CDCl_3$, 400 MHz, 298 K).

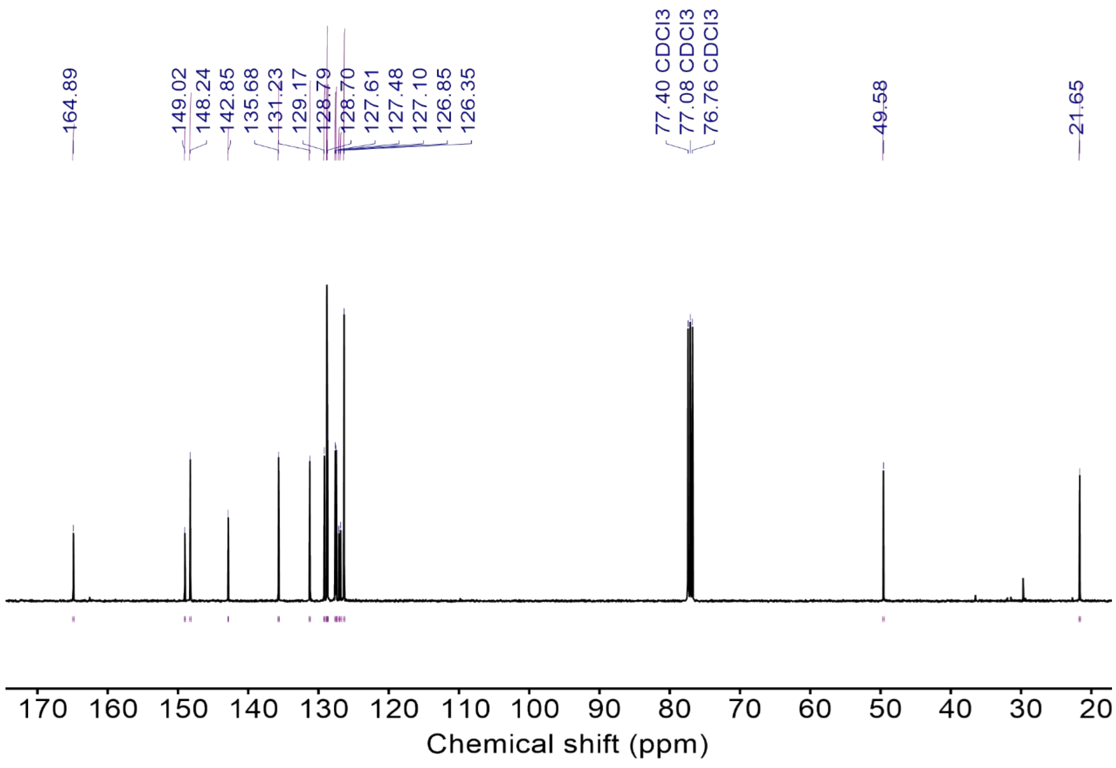

^{13}C NMR spectrum of $\mathbf{3^{\mathit{R}}MB}$ ($CDCl_3$, 101 MHz, 298 K).

3^{R}Nea

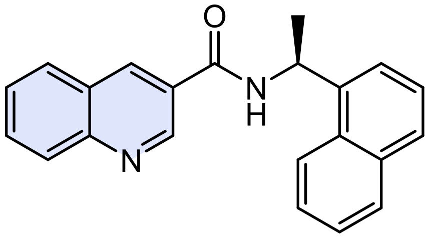

Purified by column chromatography on a silica gel column using dichloromethane and petroleum ether as the eluent to give the compound as white powder (1.39 g, 85% yield). ^{1}H NMR (400 MHz, Chloroform-*d*) δ 9.22 (d, *J* = 2.2 Hz, 1H), 8.52 (d, *J* = 2.2 Hz, 1H), 8.17 (d, *J* = 8.4 Hz, 1H), 8.12 – 8.05 (m, 1H), 7.84 (dd, *J* = 8.1, 1.5 Hz, 1H), 7.81 – 7.72 (m, 3H), 7.64 – 7.40 (m, 5H), 6.90 (d, *J* = 7.9 Hz, 1H), 6.16 (p, *J* = 7.0 Hz, 1H), 1.82 (d, *J* = 6.8 Hz, 3H). ^{13}C NMR (101 MHz, Chloroform-*d*) δ 164.64, 149.07, 148.20, 137.80, 135.58, 133.96, 131.22, 131.17, 129.21, 128.88, 128.71, 128.64, 127.45, 126.85, 126.80, 126.78, 125.99, 125.20, 123.28, 122.86, 45.48, 20.64. HRMS (TOF) m/z [M+H]$^+$: calcd for $C_{22}H_{19}N_2O^+$ at 327.1497, found at 327.1492.

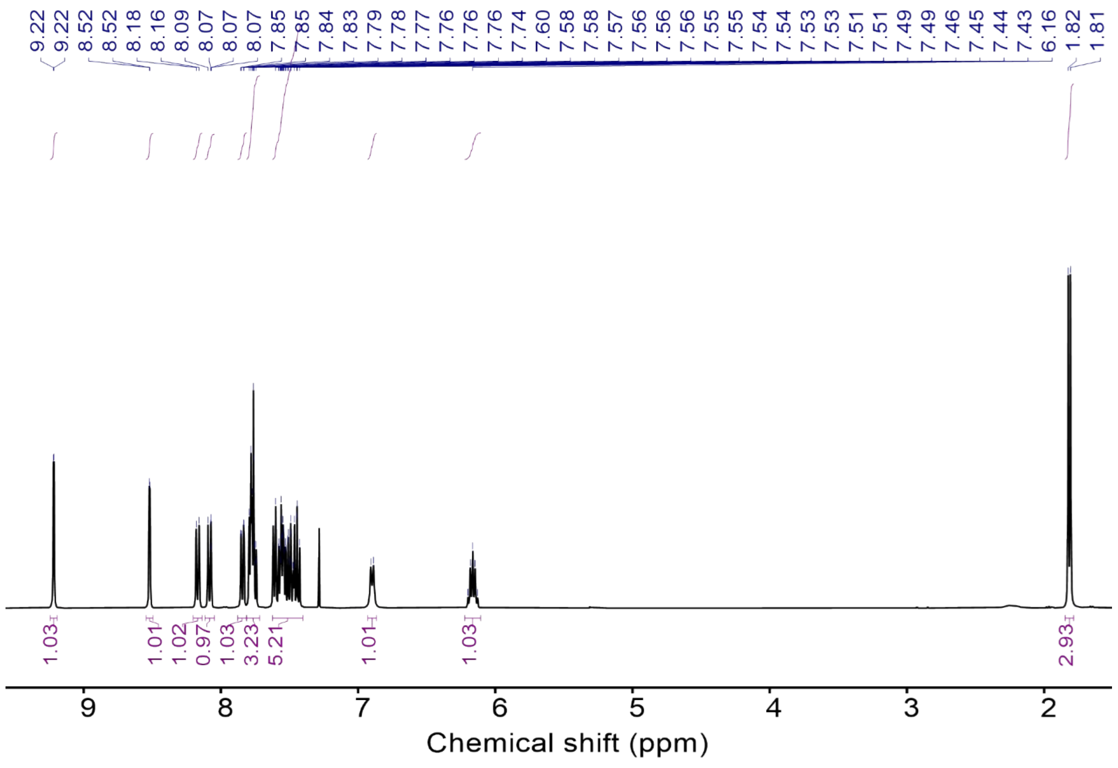

^{1}H NMR of **3^{R}Nea** ($CDCl_3$, 400 MHz, 298 K).

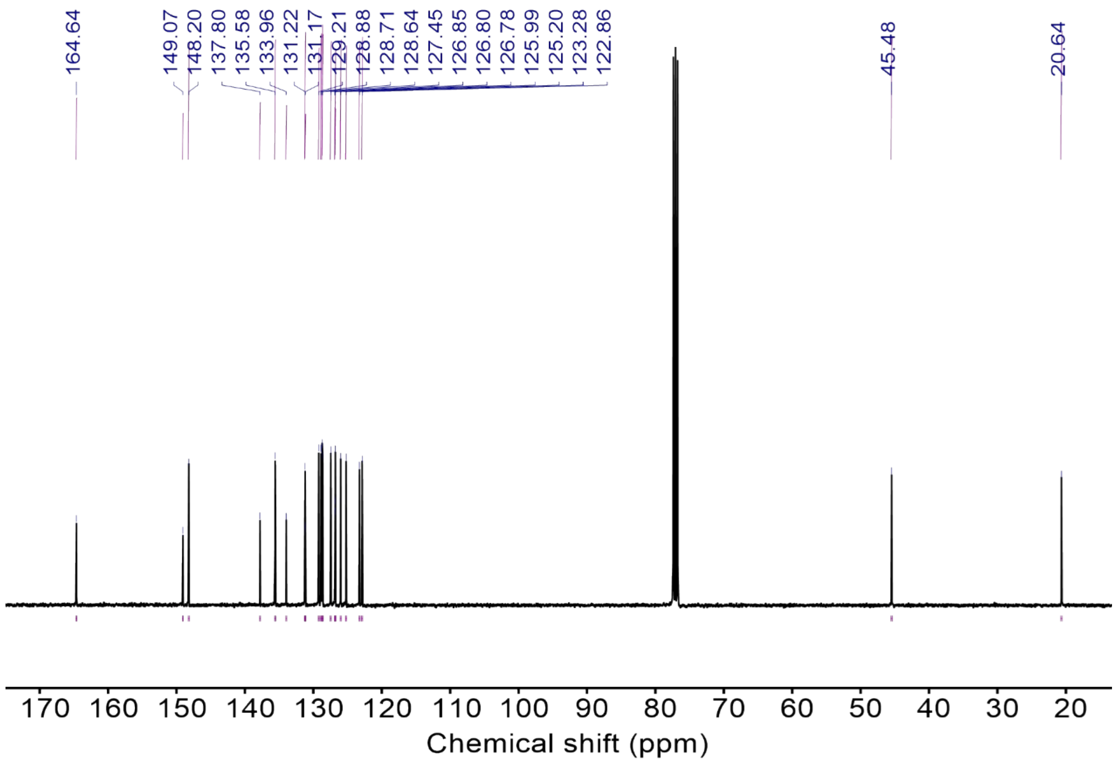

^{13}C NMR spectrum of **3^{R}Nea** ($CDCl_3$, 101 MHz, 298 K).

3^{R}Nea-I

65% yield, yellow powder. ^{1}H NMR (400 MHz, Chloroform-*d*) δ 9.32 (s, 1H), 9.07 (s, 1H), 8.20 (d, *J* = 8.5 Hz, 1H), 8.06 (d, *J* = 8.2 Hz, 1H), 8.00 – 7.94 (m, 1H), 7.94 – 7.86 (m, 2H), 7.74 (t, *J* = 7.6 Hz, 1H), 7.69 – 7.63 (m, 2H), 7.60 (d, *J* = 8.1 Hz, 1H), 7.45 – 7.34 (m, 3H), 5.95 (q, *J* = 7.0 Hz, 1H), 1.72 (d, *J* = 6.8 Hz, 3H).

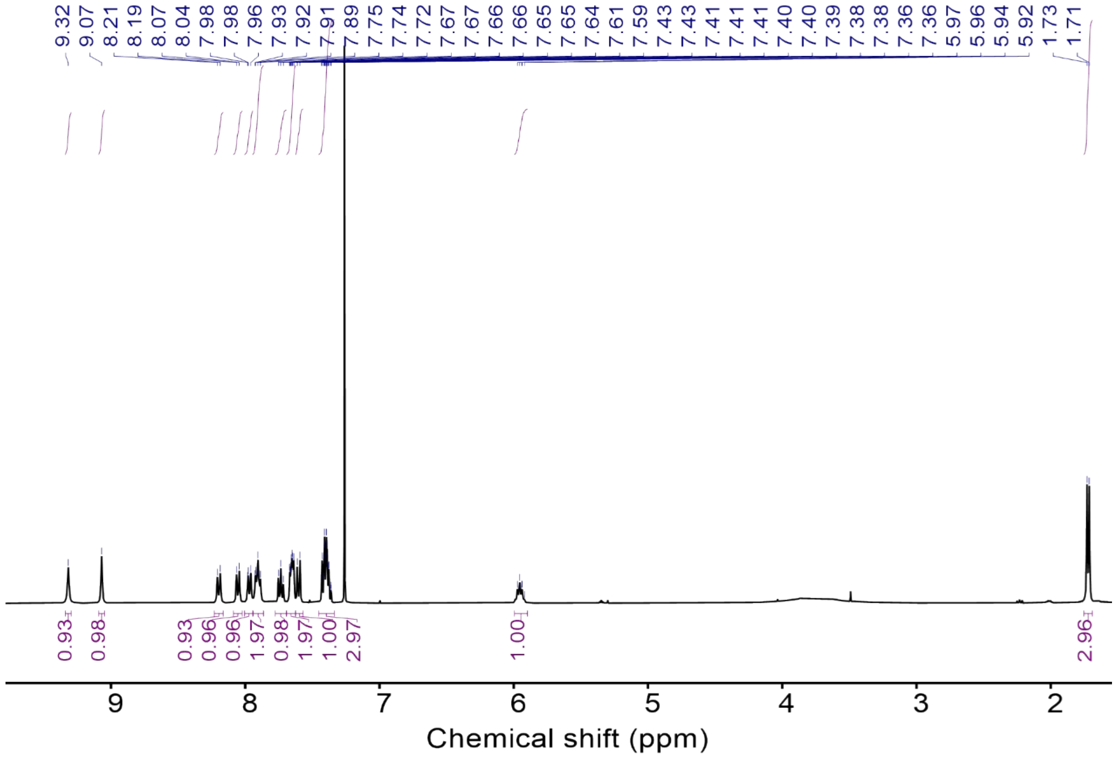

^{1}H NMR spectrum of **3[R]Nea-I** ($CDCl_3$, 400 MHz, 298 K).

3[R]MNea

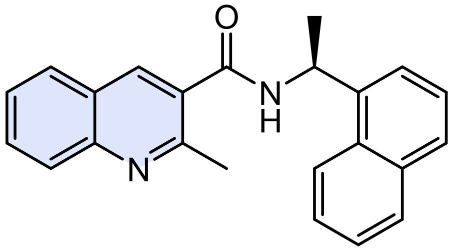

Purified by column chromatography on a silica gel column using dichloromethane and petroleum ether as the eluent to give the compound as white powder (1.36 g, 80% yield). ^{1}H NMR (400 MHz, Chloroform-*d*) δ 8.28 – 8.22 (m, 1H), 7.93 – 7.87 (m, 3H), 7.83 (d, *J* = 8.2 Hz, 1H), 7.69 – 7.57 (m, 4H), 7.54 (ddd, *J* = 8.0, 6.8, 1.3 Hz, 1H), 7.50 – 7.39 (m, 2H), 6.35 (s, 1H), 6.23 – 6.11 (m, 1H), 2.77 (d, *J* = 1.8 Hz, 3H), 1.84 (d, *J* = 6.7 Hz, 3H). ^{13}C NMR (101 MHz, Chloroform-*d*) δ 167.49, 156.21, 147.64, 137.59, 134.33, 134.03, 131.22, 130.61, 130.13, 128.96, 128.73, 128.41, 127.68, 126.79, 126.48, 126.10, 125.48, 125.23, 123.36, 122.79, 45.17, 23.71, 20.51. HRMS (TOF) m/z $[M+H]^+$: calcd for $C_{23}H_{21}N_2O^+$ at 341.1654, found at 341.1660.

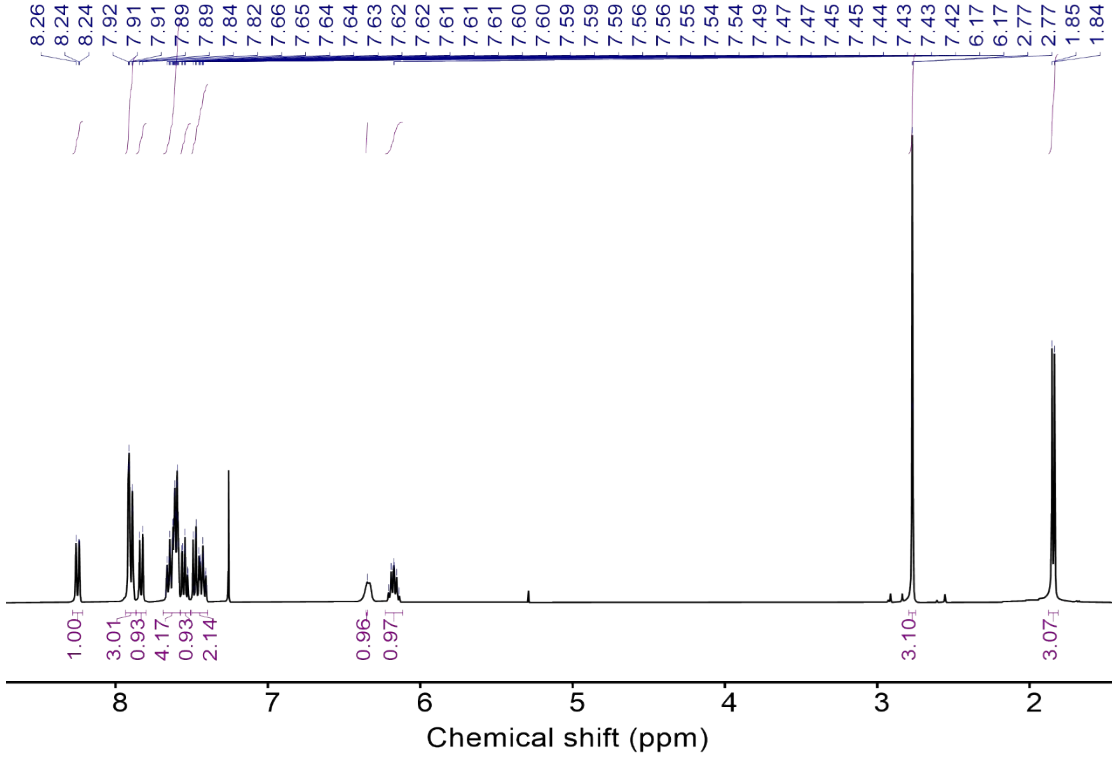

^{1}H NMR spectrum of **3^{R}MNea** ($CDCl_3$, 400 MHz, 298 K).

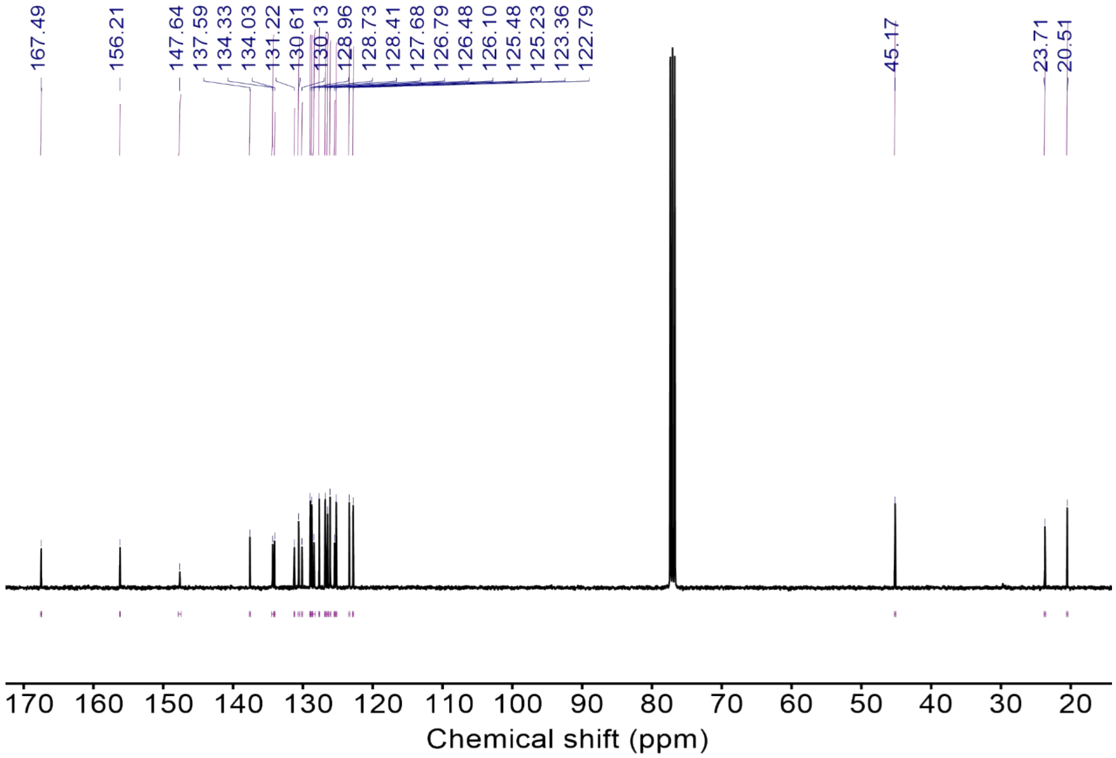

^{13}C NMR spectrum of **3^{R}MNea** ($CDCl_3$, 101 MHz, 298 K).

3^{R}MNea-I

68% yield, yellow powder. ^{1}H NMR (400 MHz, Chloroform-*d*) δ 8.59 (s, 1H), 8.24 – 8.17 (m, 1H), 8.06 (d, *J* = 8.5 Hz, 1H), 7.93 (dd, *J* = 8.3, 1.3 Hz, 1H), 7.88 (dd, *J* = 7.9, 1.6 Hz, 1H), 7.81 – 7.73 (m, 2H), 7.67 – 7.59 (m, 2H), 7.60 – 7.39 (m, 4H), 6.12 (p, *J* = 7.0 Hz, 1H), 2.82 (s, 3H), 1.78 (d, *J* = 6.9 Hz, 3H).

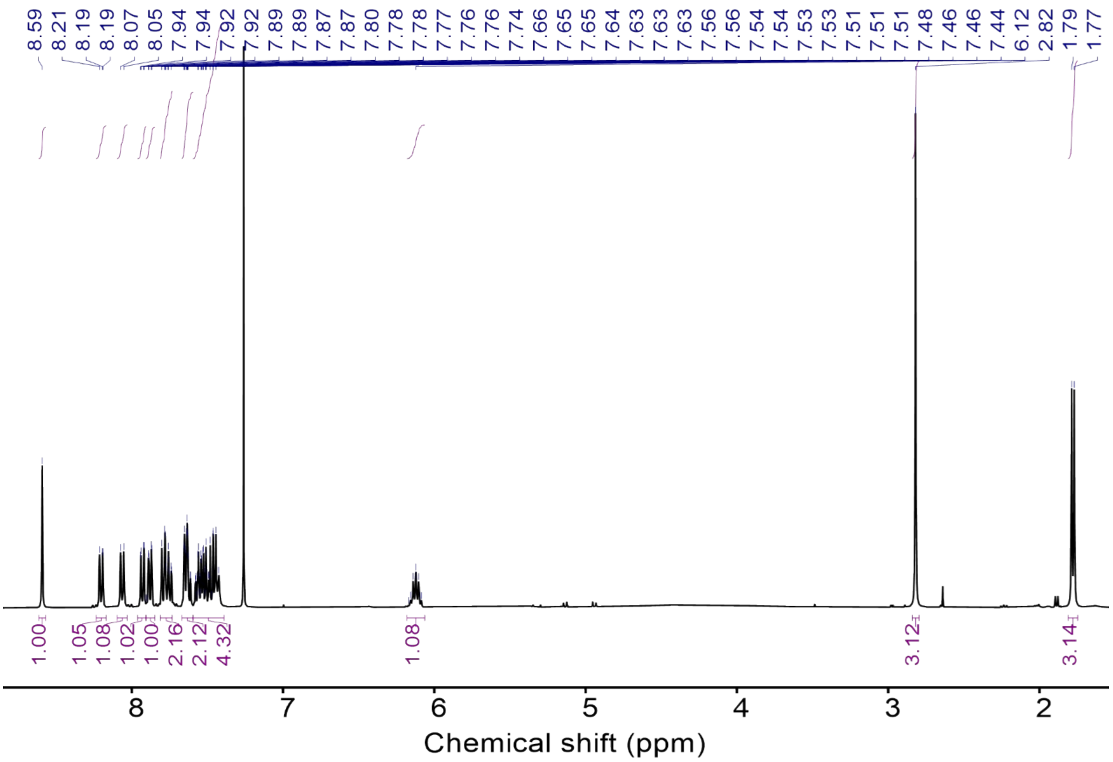

^{1}H NMR spectrum of **3^{R}MNea-I** ($CDCl_3$, 400 MHz, 298 K).

3^{L}Phe

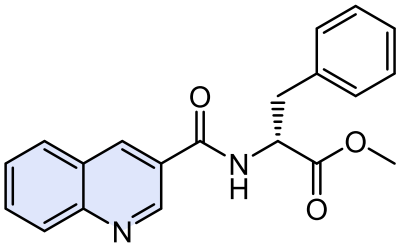

Purified by column chromatography on a silica gel column using dichloromethane and petroleum ether as the eluent to give the compound as a white solid (1.50 g, 90% yield). ^{1}H NMR (400 MHz, Chloroform-*d*) δ 9.20 (d, *J* = 2.2 Hz, 1H), 8.50 (dd, *J* = 2.3, 0.8 Hz, 1H), 8.13 (dd, *J* = 8.5, 1.1 Hz, 1H), 7.88 – 7.75 (m, 2H), 7.60 (ddd, *J* = 8.1, 6.9, 1.2

Hz, 1H), 7.35 – 7.20 (m, 3H), 7.20 – 7.13 (m, 2H), 6.91 (d, *J* = 7.6 Hz, 1H), 5.16 (dt, *J* = 7.6, 5.8 Hz, 1H), 3.80 (s, 3H), 3.40 – 3.21 (m, 2H). ^{13}C NMR (101 MHz, Chloroform-*d*) δ 172.01, 165.14, 149.28, 148.11, 135.79, 135.69, 131.44, 129.32 (d, *J* = 2.8 Hz), 128.78 (d, *J* = 4.7 Hz), 127.59, 127.37, 126.81, 126.44, 53.69, 52.61, 37.83. HRMS (TOF) m/z $[M+H]^+$: calcd for $C_{20}H_{19}N_2O_3^+$ at 335.1396, found at 335.1402.

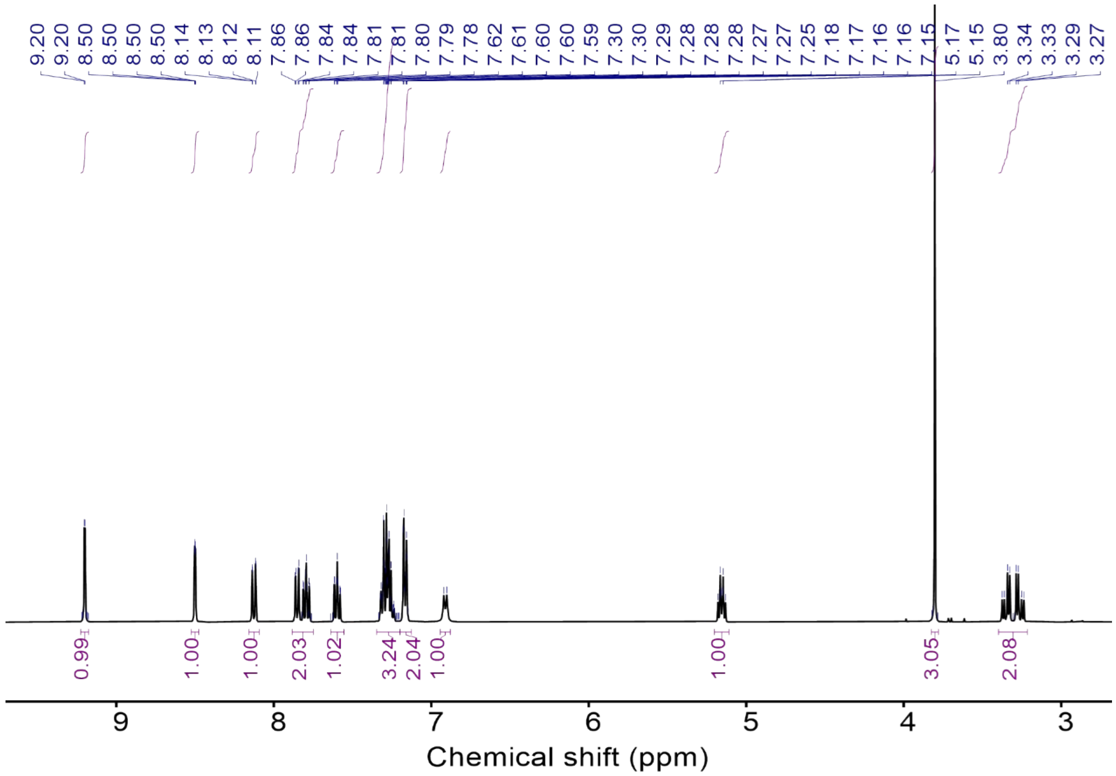

^{1}H NMR spectrum of **3^{L}Phe** (CDCl$_3$, 400 MHz, 298 K).

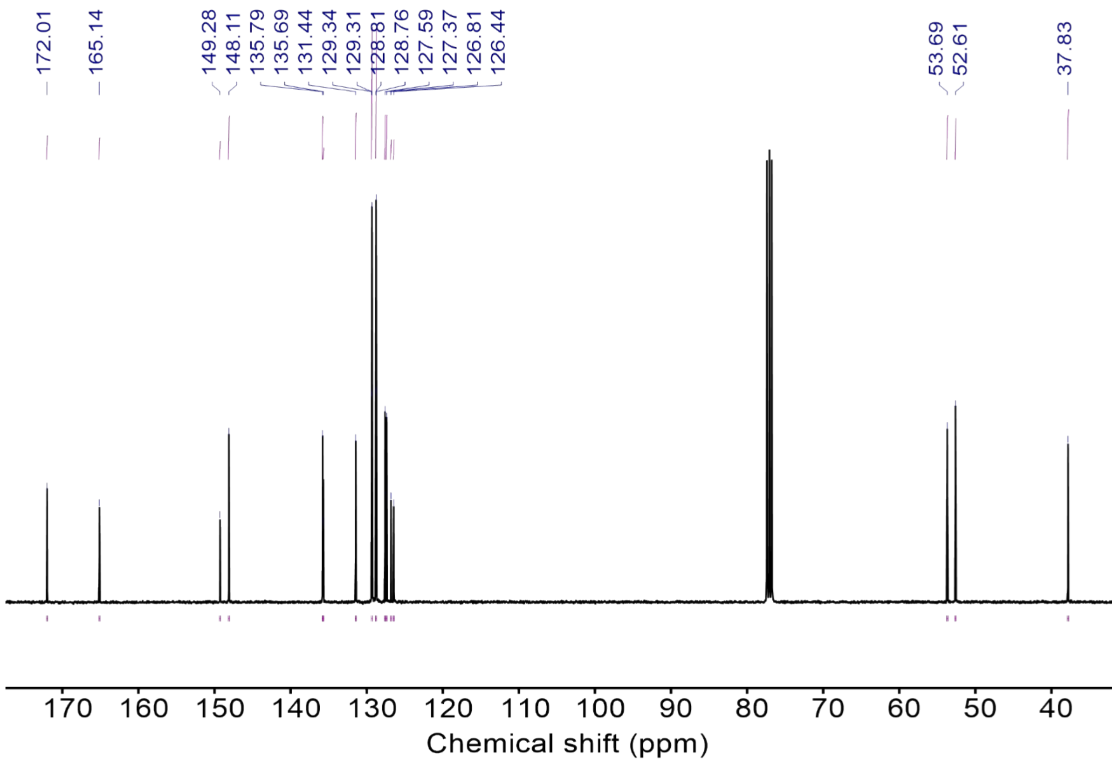

^{13}C NMR spectrum of **3*L*Phe** ($CDCl_3$, 101 MHz, 298 K).

3*L*Phe-I

68% yield, yellow powder. ^1H NMR (400 MHz, Chloroform-*d*) δ 9.38 (d, J = 2.0 Hz, 1H), 9.15 (d, J = 2.0 Hz, 1H), 8.28 (d, J = 8.6 Hz, 1H), 8.15 (dd, J = 8.4, 1.3 Hz, 1H), 8.06 – 7.97 (m, 1H), 7.82 (ddd, J = 8.2, 6.9, 1.0 Hz, 1H), 7.73 (t, J = 6.7 Hz, 1H), 7.32 – 7.19 (m, 4H), 7.19 (dt, J = 6.2, 3.1 Hz, 1H), 5.06 (td, J = 8.0, 5.6 Hz, 1H), 3.75 (s, 3H), 3.35 (dd, J = 14.0, 5.6 Hz, 1H), 3.21 (dd, J = 14.0, 8.1 Hz, 1H).

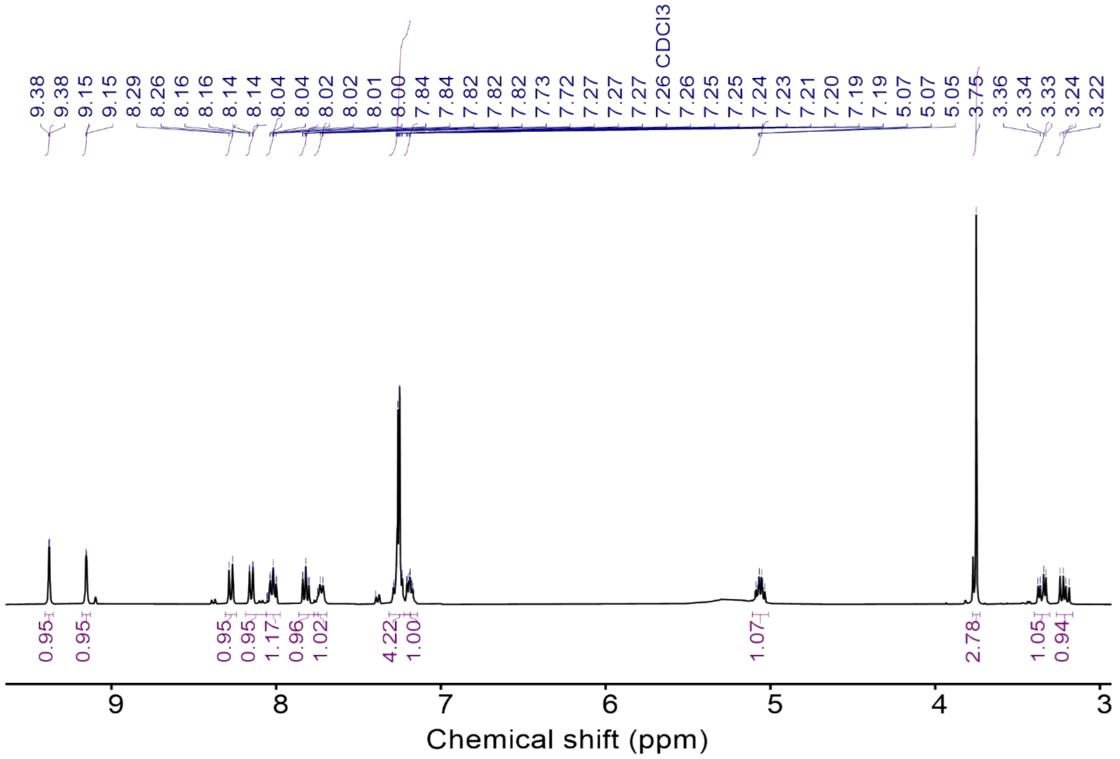

^{1}H NMR spectrum of **3^{L}Phe-I** ($CDCl_3$, 400 MHz, 298 K)

2. Additional experimental data

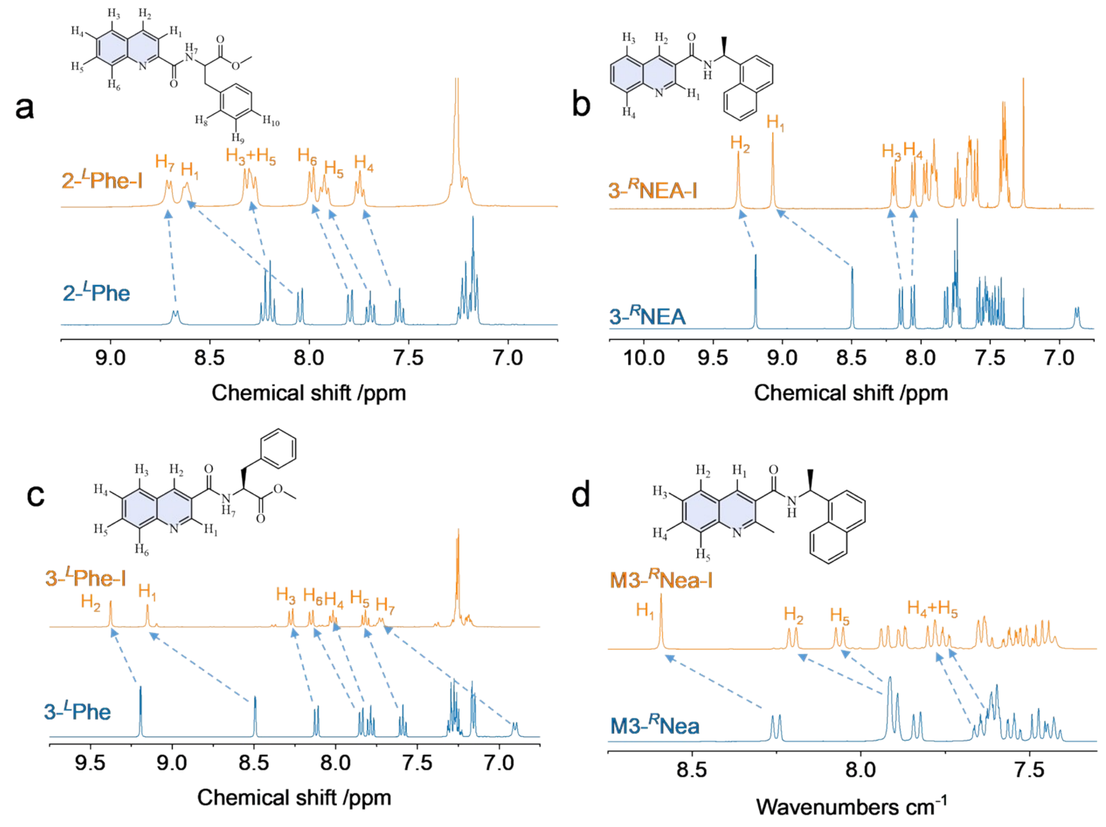

Figure S1. Aromatic region of ^{1}H NMR spectra of I-complexes and monomers ($CDCl_3$,

400 MHz, 298 K, c = 10 mM)

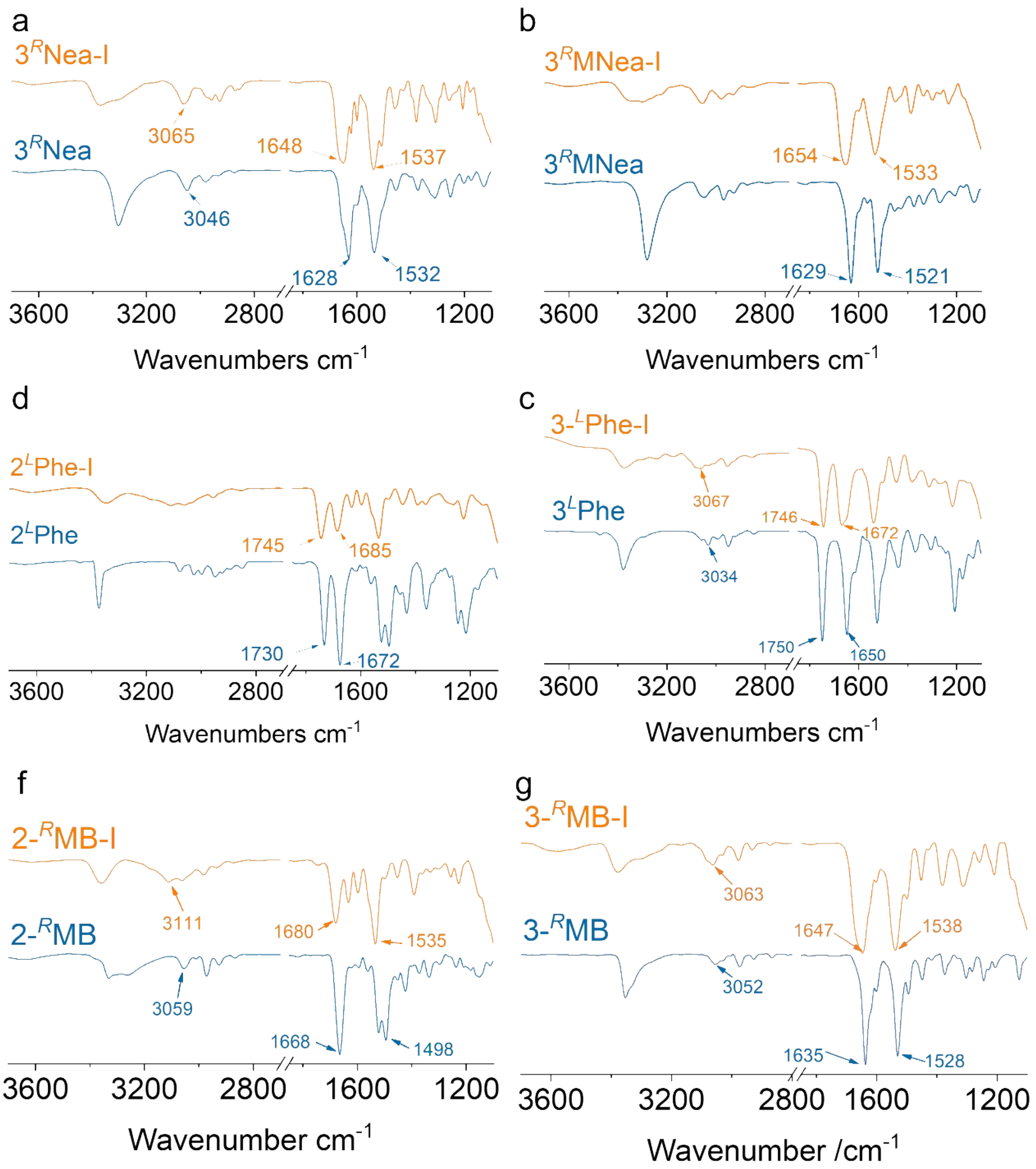

Figure S2. FITR spectra of I-complexes and monomers (KBr tablets).

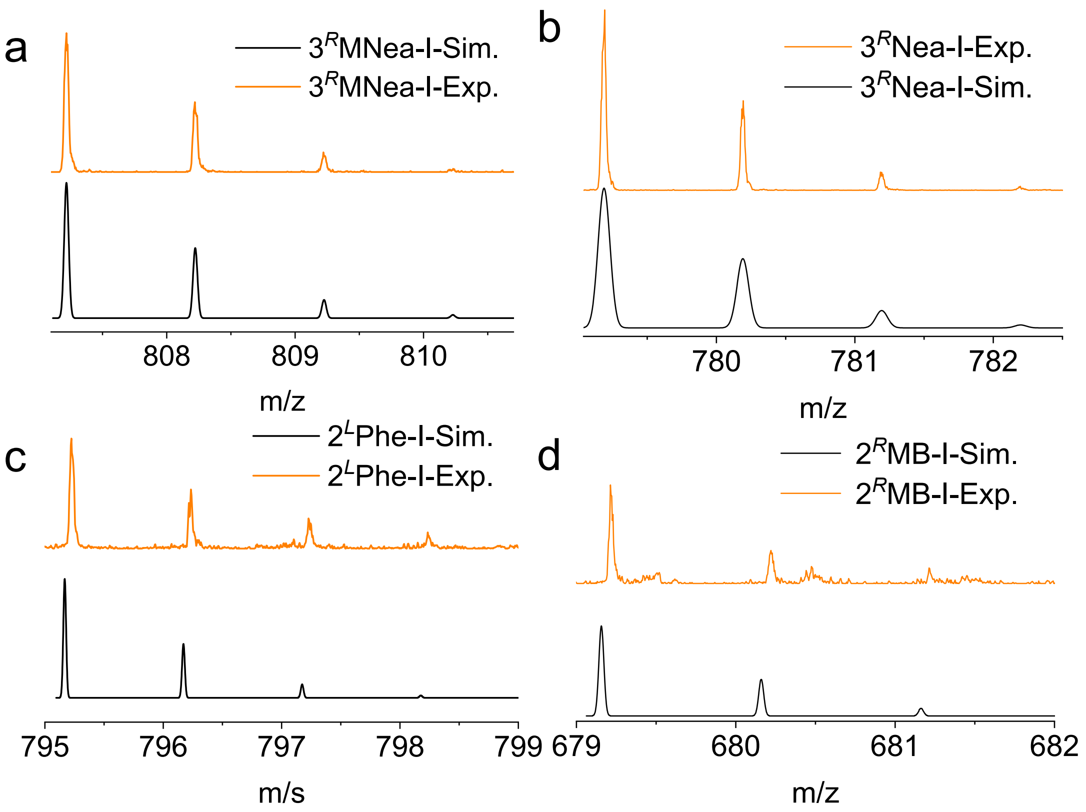

Figure S3. HRMS spectra of I-complexes.

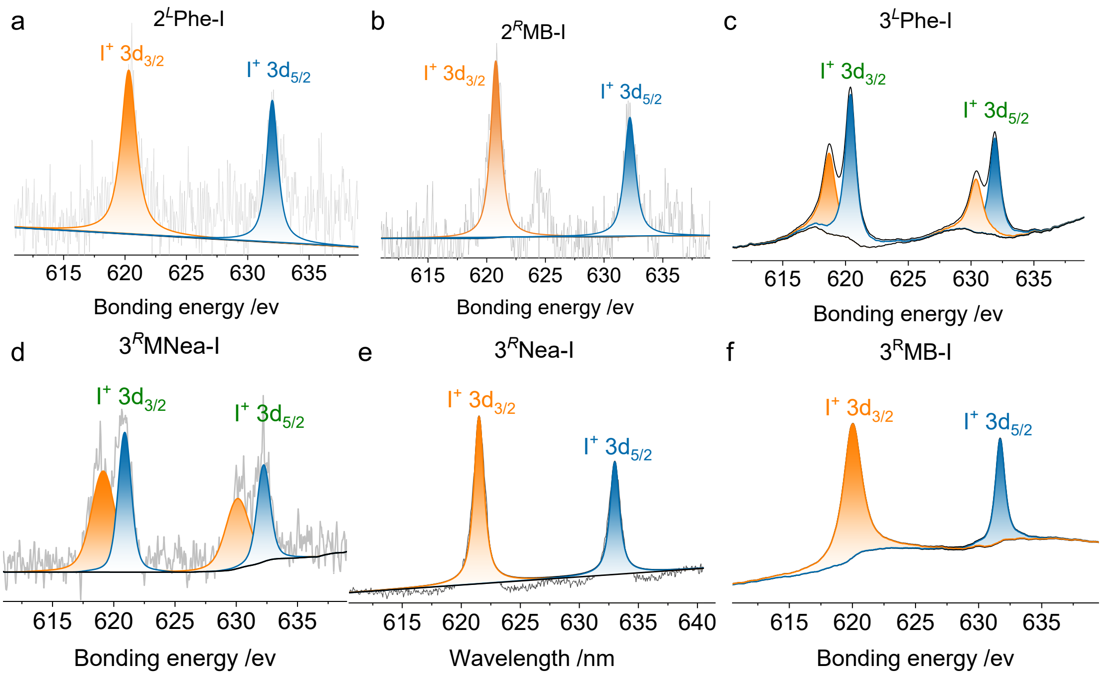

Figure S4. XPS spectra of I-complexes.

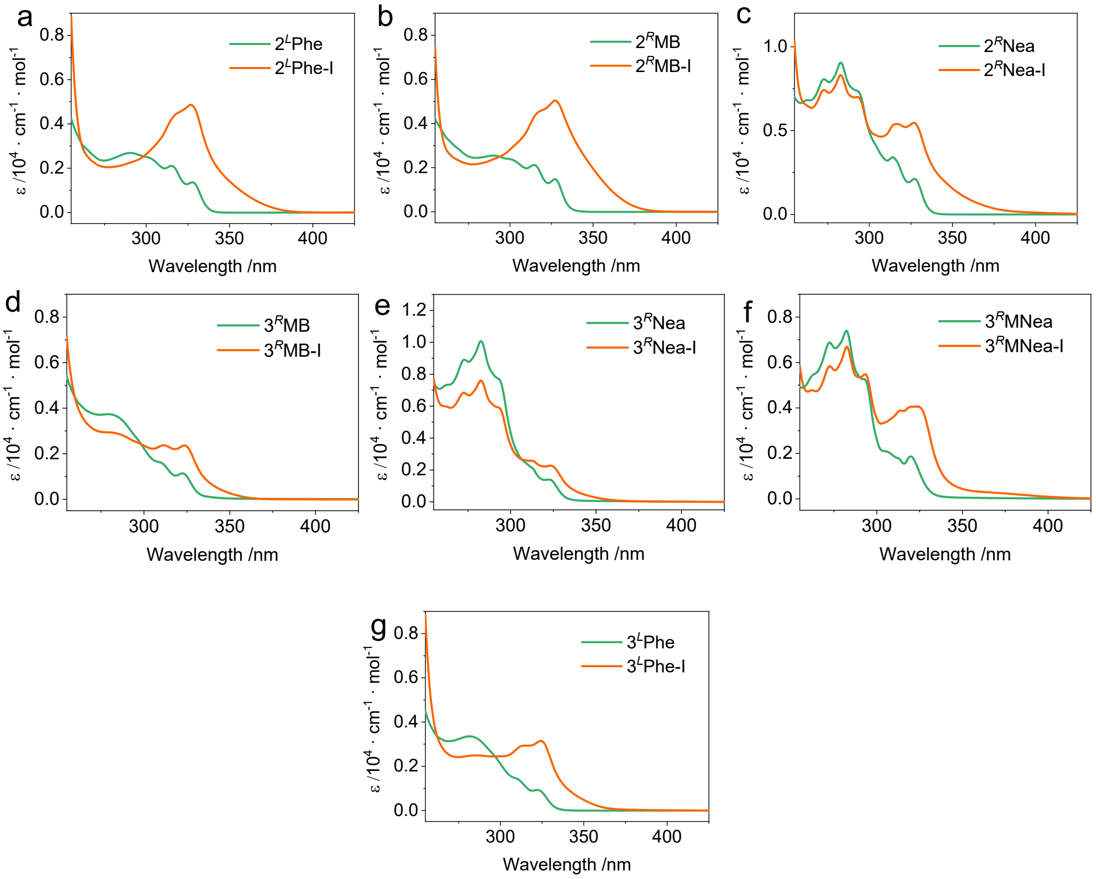

Figure S5. UV-vis absorption spectra of quinoline derivatives in DCM (0.1 mM, path length = 10 mm).

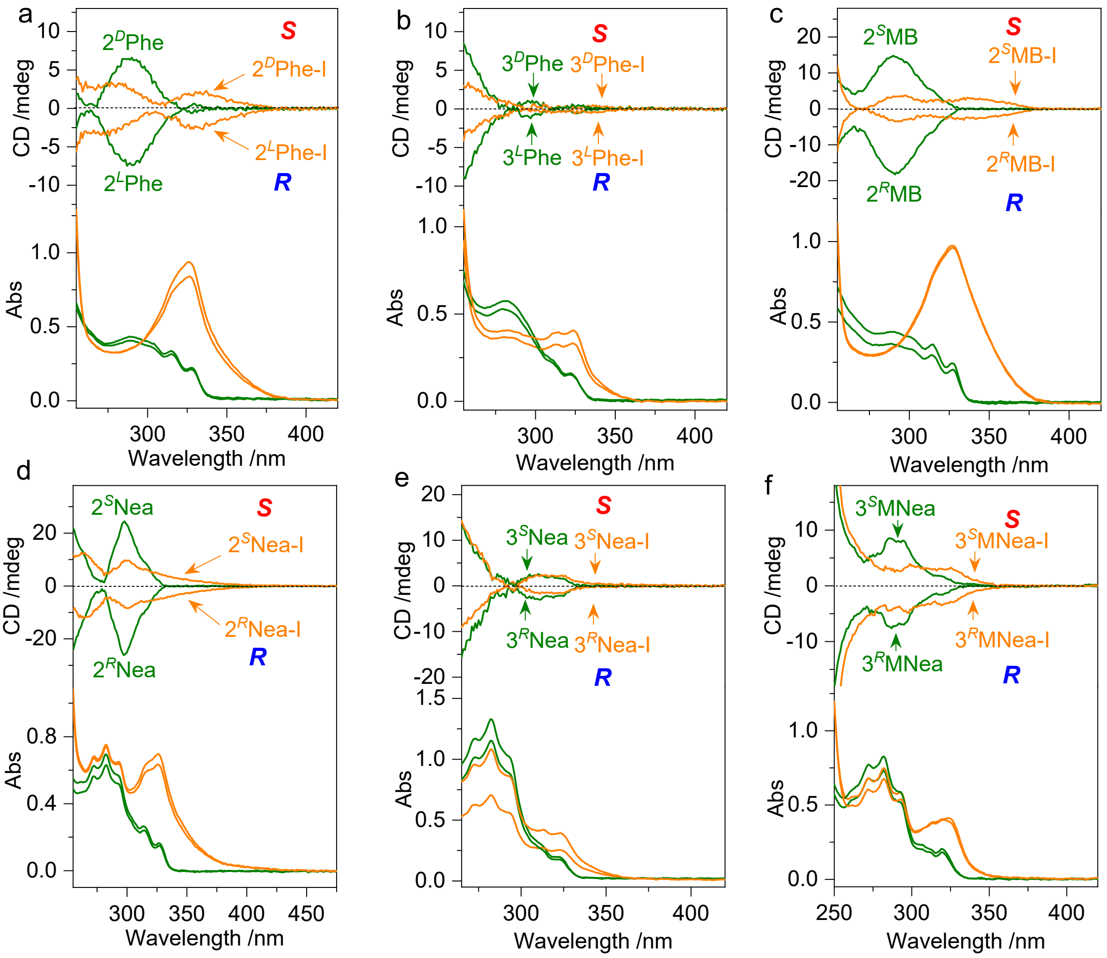

Figure S6. CD spectra of different starting compounds and halogen bonded complexes in solutions (1 mM in CH_2Cl_2, path length = 1 mm).

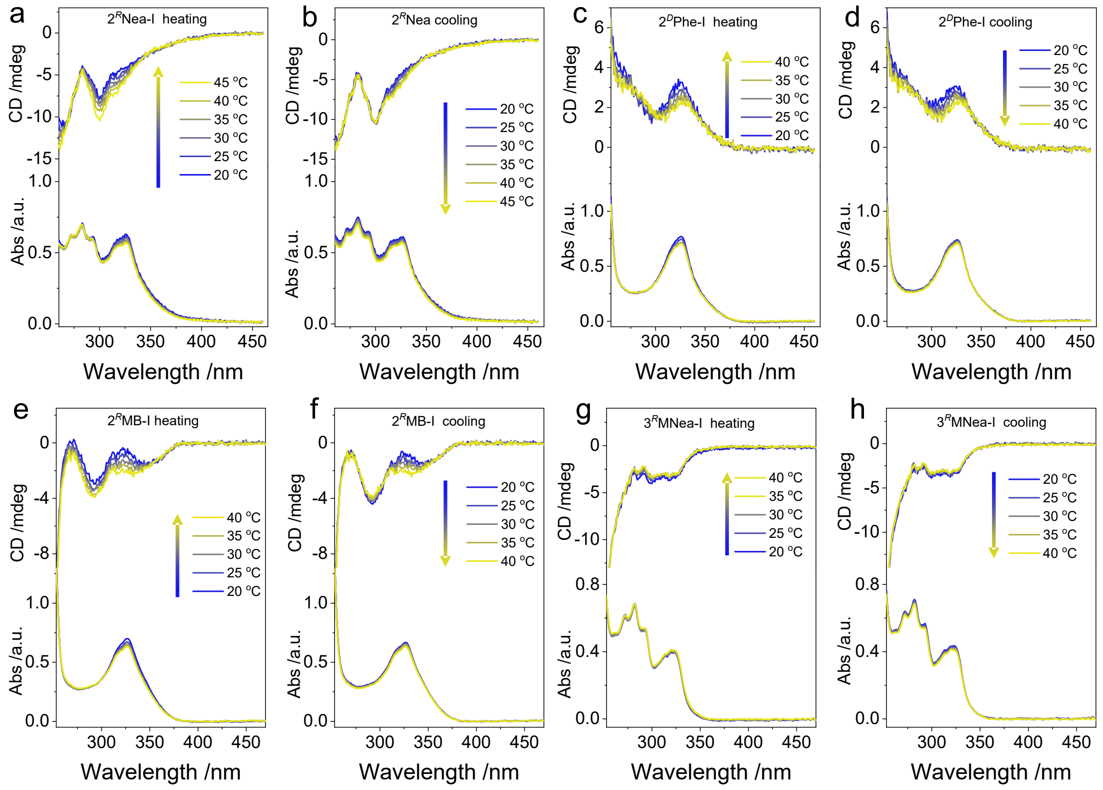

Figure S7. Temperature-variable CD spectra of 2′ substituted I-complexes (1 mM in CH_2Cl_2)

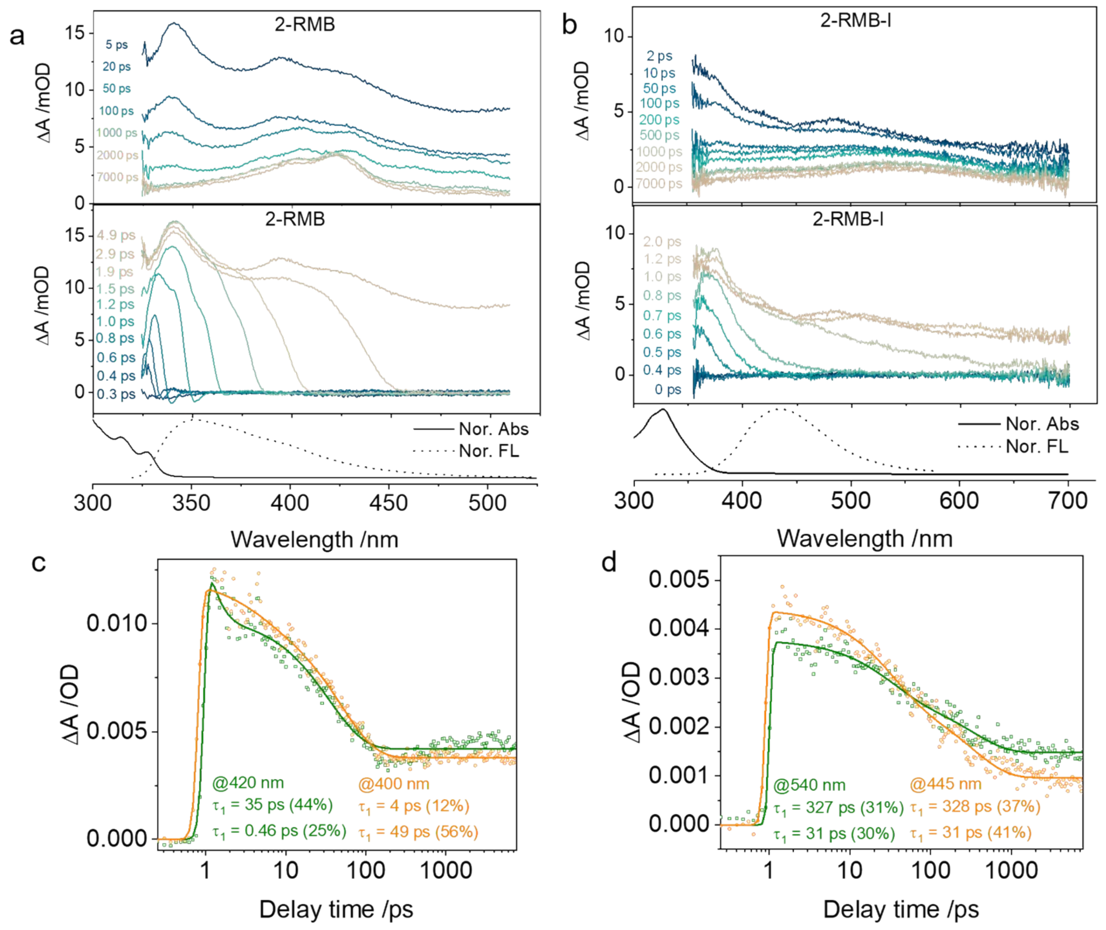

Figure S8. a, b) The TA spectra of 2^RMB and 2^RMB-I at different delay time (10 mM in CH_2Cl_2). c, d) The TA kinetic traces of 2^RMB and 2^RMB-I at specific probe wavelengths.

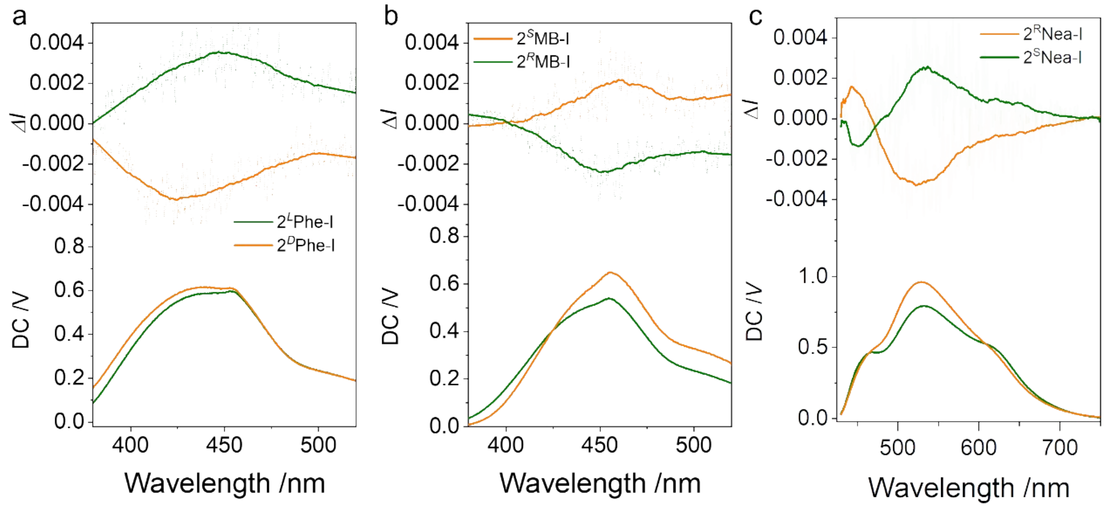

Figure S9. CPL spectra of 2′ substituted I-complexes.

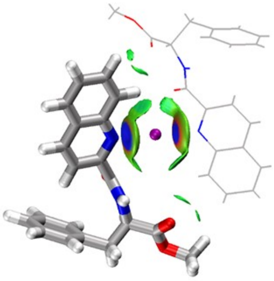

2^{L}Phe-I

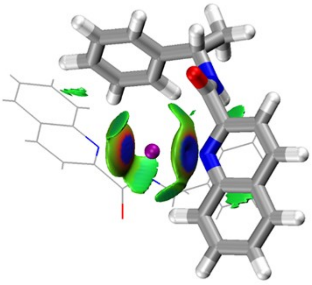

2^{R}MB-I

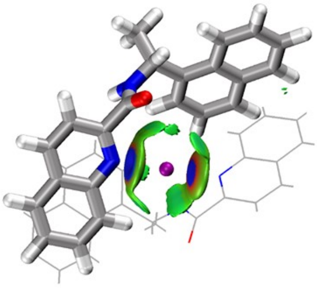

2^{R}Nea-I

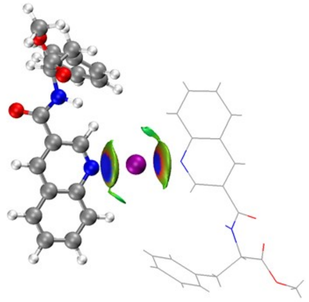

3^{L}Phe-I

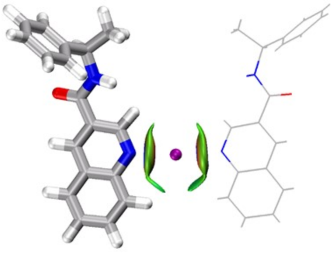

3^{R}MB-I

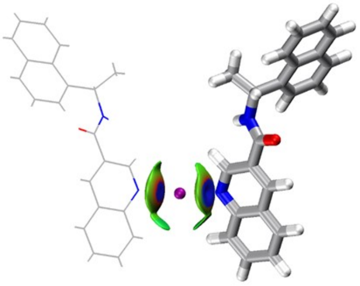

3^{R}Nea-I

Figure S10. The IGMH analysis of I-complexes (isosurface = 0.008 a.u.)

Table S1. The photographs of I-complexes and monomers under daylight and 365 nm irradiation light.

**Samples**	**Daylight**	**365 nm**
2^{R}MB	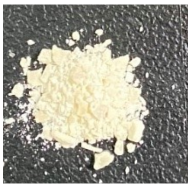	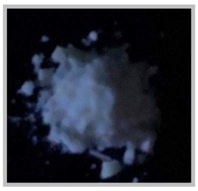
2^{R}MB-I	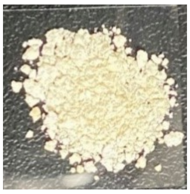	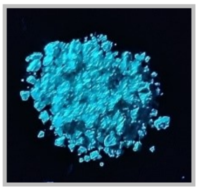
2^{L}Phe	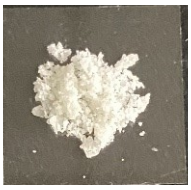	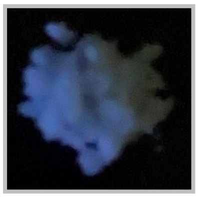

2^{L}Phe-I	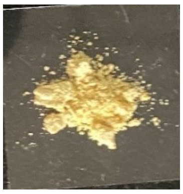	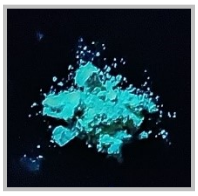
2^{R}Nea	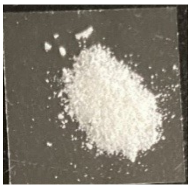	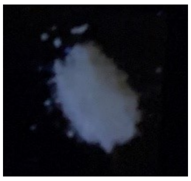
2^{R}Nea-I	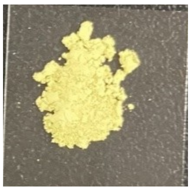	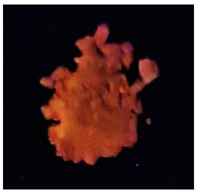
3^{R}MNea	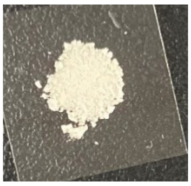	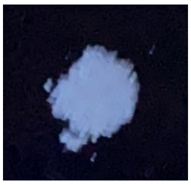
3^{R}MNea-I	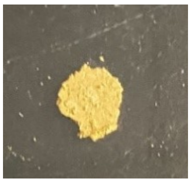	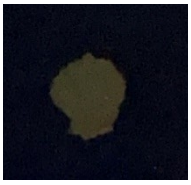
3^{R}MB	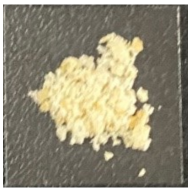	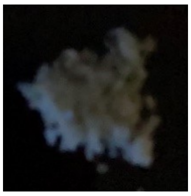
3^{R}MB-I	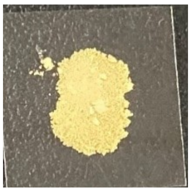	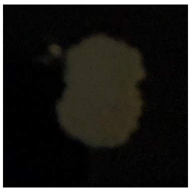
3^{L}Phe	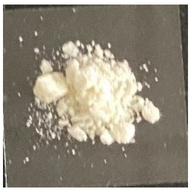	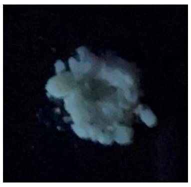

3^{L}Phe-I	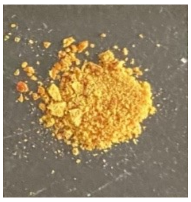	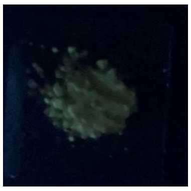

Table S2. HOMO-LUMO energy levels and gaps

	**HOMO /ev**	**LUMO /ev**	**gap /ev**
2^{R}MB	-6.60	-1.92	4.67
2^{R}MB-I	-7.06	-2.62	4.44
3^{R}MB	-6.60	-1.92	4.68
3^{R}MB-I	-6.96	-2.80	4.17
2^{L}Phe	-6.60	-1.95	4.66
2^{L}Phe-I	-7.02	-2.64	4.38
3^{L}Phe	-6.66	-1.95	4.71
3^{L}Phe-I	-7.06	-2.80	4.27
2^{R}Nea	-5.99	-1.93	4.06
2^{R}Nea-I	-6.23	-2.62	3.61
3^{R}Nea	-6.01	-1.93	4.08
3^{R}Nea-I	-6.17	-2.80	3.37

Table S3. Lifetime and quantum yield of I-complexes and monnmers.

	Lifetime[a] /ns	QY[a] /%	QY[b] /%
2^{R}MB	**1.2**		**0.28**
2^{R}MB-I	**11.5**	**1.53**	**3.96**
2^{L}Phe	**3.9**	**0.62**	**0.62**
2^{L}Phe-I	**14.3**	**2.51**	**5.17**
2^{R}Nea	**2.6**	**0.24**	**0.77**
2^{R}Nea-I	**717.5**	**0.38**	**0.98**
3 ^{R}MNea-I	**1.8**	**0.12**	
3^{L}Phe	**0.3**	**0.24**	

3^LPhe-I	**16.5**	**0.24**
3^RNea	**2.7**	**0.27**
3^RNea-I	**15.6**	**1.62**

a. solid phase; b. in PMMA film.

Table S4. Crystal structure information of 3^LVal-Ag.

Deposition Number	2258561
Formula	$C_{26}H_{72}Ag_2B_2F_8N_8O_{12}$
Temperature(K)	173
Wavelength	1.54184Å
Crystal system	monoclinic
Space group	C2 (5)
a,b,c/Å	a 35.37(11) b 7.14(2) c 28.54(6)
V, $Å^3$	7116.21
Cell angles	α 90 β 90 (2) γ 90
Z, Z'	Z: 4 Z': 0
R-factor (%)	7.64

3. Optimized geometries

Computational details: All the geometries were optimized with Gaussian 16 program, Revision A.03[3] at the B3LYP-D3 def2TZVP level of theory with Grimme D3 dispersion correction (BJ-damping) in dichloromethane using SMD solvent model.

2^RMB-I

I	-0.03476400	-0.08955300	1.09549200
N	-2.14420600	0.86430100	1.07373500
O	-0.49551700	3.57631100	0.68978300
N	0.65906900	-2.55245600	-1.48721100
H	1.17235300	-1.80700800	-1.93490800
N	2.07418300	-1.02208700	0.97480800
O	0.40411600	-3.67884500	0.48304100
N	-0.71590500	2.48361900	-1.30014100
H	-1.21988100	1.74724200	-1.77672600
C	-4.21248500	-1.67060800	2.73135800
H	-4.10100400	-2.63503600	3.20916600
C	-3.23326300	0.21948200	1.60687800
C	4.45807600	-0.97783500	1.37728900
C	3.16583300	-0.39663800	1.52628200

C	2.21247400	-2.15934500	0.31227000
C	-5.64476300	0.12698800	2.01926800
H	-6.61974900	0.58793400	1.92662400
C	5.58024300	-0.31679500	1.92650800
H	6.55606800	-0.76857600	1.80250500
C	0.42719300	3.10211300	-1.97649100
H	0.56716900	4.06970900	-1.49813700
C	-4.52367700	0.81317600	1.49925400
C	-3.10282000	-1.03265300	2.23768700
H	-2.12875500	-1.49090800	2.31664000
C	3.03639100	0.82103000	2.22120700
H	2.06253100	1.27360900	2.32831800
C	0.97935900	-2.86098300	-0.22298200
C	4.57476400	-2.18816600	0.66716700
H	5.55213700	-2.63680500	0.54274000
C	3.46009700	-2.77828300	0.13820000
H	3.51166300	-3.70748000	-0.41096900
C	-0.52532800	-3.07370400	-2.17732300
H	-0.84592300	-3.93553800	-1.59653100
C	-2.28132900	2.03727900	0.47777600
C	1.82400300	1.00670300	-2.29306800
H	0.98650500	0.54850500	-2.80386200
C	-3.76053400	-1.30257100	-1.23940200
H	-4.59275200	-1.46190700	-0.56674600
C	5.42884900	0.86815600	2.59553900
H	6.28937800	1.37378000	3.01292100
C	-5.49357100	-1.09329100	2.62192100
H	-6.35349200	-1.61802200	3.01630100
C	-2.72840300	-2.23314900	-1.29338900
H	-2.76063300	-3.11187200	-0.66116700
C	-1.60727900	-0.90599100	-2.94874300
H	-0.78007200	-0.74358900	-3.62751500
C	-1.05269700	2.76164200	-0.03484500
C	-3.52597700	2.67448300	0.35410300
H	-3.57651700	3.63326500	-0.14155700
C	1.70296200	2.30100600	-1.78611200
C	4.14697700	1.43496000	2.74184600
H	4.03560300	2.37397200	3.26778200
C	-1.64492100	-2.04921300	-2.14901100
C	0.08360600	3.31225600	-3.44736100
H	-0.09201500	2.36061500	-3.95188000
H	-0.81156900	3.92858400	-3.54405100
H	0.90928000	3.81316500	-3.95295900
C	3.01209600	0.30021700	-2.15316500

H	3.10023900	-0.69911300	-2.56233300
C	-3.71687800	-0.16854600	-2.03971200
H	-4.51714800	0.55841600	-1.99969100
C	-0.13953000	-3.52762700	-3.57996200
H	0.61165900	-4.31666200	-3.52375600
H	-1.01497000	-3.91536600	-4.10172800
H	0.27072800	-2.70571900	-4.16949200
C	-2.63614600	0.02701700	-2.89565400
H	-2.60186600	0.90386100	-3.53112900
C	4.09610200	0.87689000	-1.49810800
H	5.02122000	0.32733200	-1.38900500
C	2.78895900	2.86504000	-1.12252600
H	2.70118600	3.86486600	-0.71507200
C	-4.63955500	2.06200200	0.85848500
H	-5.61533900	2.52156800	0.76643000
C	3.97986200	2.15970500	-0.97926800
H	4.81352700	2.61162300	-0.45770200

2^{R}Nea-I

I	-0.00063800	0.00090200	-1.20305100
O	1.06221000	-3.36836800	-1.32300900
O	-1.06154500	3.37297300	-1.32199800
N	-1.57600300	-1.69326300	-1.12248300
N	-0.61694500	3.00627000	0.88695500
H	0.11268100	2.75933400	1.53972300
N	1.57524800	1.69410400	-1.12197500
N	0.61720400	-3.00610700	0.88651600
H	-0.11237800	-2.76042300	1.53981200
C	3.83428300	2.52774300	-1.34193500
C	2.88967700	1.46500900	-1.44673600
C	-1.18004100	-2.88680500	-0.70679600
C	-2.06648200	-3.96650100	-0.57679000
H	-1.68799700	-4.91789100	-0.23135600
C	5.18610000	2.28545600	-1.67684200
H	5.89238100	3.10125700	-1.59126400
C	3.38581700	3.78570100	-0.89652900
H	4.09470000	4.59929000	-0.80814900
C	-3.95586300	1.41458800	1.33233900
C	-3.83451000	-2.52849900	-1.34179600
C	4.86190200	-2.39819100	0.85693900
H	4.51672900	-3.40517300	0.67241100
C	5.58591500	1.04396200	-2.09100800
H	6.62246700	0.85801200	-2.33760800

C	4.46208200	-0.09606600	1.56533900
C	2.57733800	-1.67879300	1.58721600
C	-2.89061000	-1.46518000	-1.44708100
C	3.32889100	0.19515900	-1.86987800
H	2.62539100	-0.62026000	-1.93736300
C	1.18015100	2.88800600	-0.70650000
C	4.64895900	-0.00392900	-2.18350300
H	4.97596600	-0.98566100	-2.49834000
C	2.06734200	3.96712900	-0.57699700
H	1.68957100	4.91886000	-0.23172100
C	3.59140900	0.91435000	2.03652200
H	3.98667100	1.90971400	2.19706000
C	3.95662300	-1.41538300	1.33169100
C	-2.57662300	1.67827300	1.58788000
C	-1.99319600	3.06858800	1.37439400
H	-2.54730800	3.57331200	0.58876000
C	-5.58718600	-1.04610600	-2.09115600
H	-6.62388800	-0.86090500	-2.33768300
C	1.99355500	-3.06891100	1.37358500
H	2.54737800	-3.57359500	0.58771000
C	-4.65090800	0.00234700	-2.18420400
H	-4.97857500	0.98371000	-2.49947300
C	-4.46110600	0.09520200	1.56607200
C	-3.38515000	-3.78602900	-0.89608700
H	-4.09350300	-4.60003400	-0.80729500
C	-5.82771700	-0.17847900	1.32086900
H	-6.19147300	-1.18330600	1.49690900
C	-5.18653300	-2.28719100	-1.67657600
H	-5.89229300	-3.10340100	-1.59059500
C	0.29169000	-3.10045500	-0.41118200
C	-4.86133400	2.39720300	0.85753900
H	-4.51632500	3.40421000	0.67285900
C	2.27691500	0.63025700	2.28774300
H	1.60845200	1.39732700	2.65910000
C	-3.59026500	-0.91507900	2.03722400
H	-3.98537700	-1.91050200	2.19778400
C	-3.33068500	-0.19576600	-1.87063900
H	-2.62765000	0.62002600	-1.93860800
C	-1.77891700	0.66769800	2.06697100
H	-0.73575100	0.85979100	2.27234100
C	-2.27580800	-0.63077100	2.28839900
H	-1.60719000	-1.39772400	2.65971800
C	-0.29138000	3.10273700	-0.41057600
C	-2.04905000	3.90628500	2.65016900

H	-1.43335900	3.45540700	3.43166100
H	-3.07371600	3.96171500	3.01786700
H	-1.68991600	4.91871000	2.45862600
C	-6.18065000	2.09748700	0.63043500
H	-6.85097300	2.86617000	0.26711400
C	2.04943400	-3.90689600	2.64916100
H	3.07414800	-3.96261800	3.01667000
H	1.69005100	-4.91920900	2.45749200
H	1.43399200	-3.45603100	3.43085700
C	1.77978700	-0.66811600	2.06632800
H	0.73657100	-0.86007100	2.27156000
C	5.82870700	0.17739700	1.31998200
H	6.19263800	1.18218100	1.49591800
C	6.18124400	-2.09868400	0.62969300
H	6.85142700	-2.86750000	0.26639500
C	6.67371300	-0.79949400	0.86531100
H	7.71657100	-0.57718700	0.67875300
C	-6.67291400	0.79825100	0.86621800
H	-7.71575800	0.57577800	0.67978000

2^{L}Phe-I

C	-0.36819000	5.46281300	-2.31594300
C	0.55535600	4.40082000	-2.24977400
C	0.22156100	3.20411100	-1.66711700
C	-1.06439500	3.01916800	-1.12366500
C	-2.00132500	4.09325300	-1.17817800
C	-1.62179800	5.31170500	-1.78607200
N	-1.43058800	1.82763800	-0.54293000
C	-2.63801700	1.68031800	-0.02282200
C	-3.59802500	2.70482300	-0.04120100
C	-3.28013400	3.90242200	-0.61998000
C	-2.99874500	0.39798100	0.69634400
N	-3.73261600	-0.46278400	-0.02736300
O	-2.67555300	0.25181500	1.86519600
C	-4.30312400	-1.66808800	0.52982500
C	-3.86144600	-2.87751600	-0.27696900
O	-4.00623400	-3.99636000	0.42895800
C	-3.70339500	-5.23794100	-0.24391200
O	-3.47741900	-2.83085000	-1.42159600
C	-5.85038600	-1.60565500	0.55145500
C	-6.35571200	-0.37251400	1.25042200
C	-6.88608800	0.68965500	0.51996100
C	-7.31656800	1.84886900	1.15785000
C	-7.21580500	1.96076400	2.53965800

C	-6.68877700	0.90514100	3.27842100
C	-6.26376400	-0.25150800	2.63759200
I	0.00004700	-0.00003600	-0.53795800
N	1.43066900	-1.82770000	-0.54293400
C	2.63808400	-1.68041000	-0.02278800
C	3.59806900	-2.70493500	-0.04114500
C	3.28019700	-3.90250300	-0.61999400
C	2.00140400	-4.09330000	-1.17824600
C	1.06447600	-3.01921600	-1.12370200
C	1.62188200	-5.31172600	-1.78619000
C	0.36827400	-5.46281100	-2.31607200
C	-0.55527500	-4.40082400	-2.24985600
C	-0.22148400	-3.20413900	-1.66714700
C	2.99885900	-0.39809000	0.69640200
N	3.73247100	0.46280900	-0.02740700
O	2.67586300	-0.25202100	1.86531600
C	4.30299000	1.66813500	0.52974600
C	3.86134200	2.87752600	-0.27712500
O	4.00607200	3.99639300	0.42877600
C	3.70325700	5.23795500	-0.24414100
O	3.47736100	2.83081400	-1.42176400
C	5.85024200	1.60570300	0.55141500
C	6.35559200	0.37260300	1.25043700
C	6.88588400	-0.68964600	0.52003800
C	7.31639300	-1.84880500	1.15801500
C	7.21574600	-1.96055200	2.53984000
C	6.68880500	-0.90484300	3.27854300
C	6.26376100	0.25174600	2.63763000
H	-0.08074500	6.39595900	-2.78190600
H	1.54967200	4.52599900	-2.65569200
H	0.94933600	2.40999200	-1.61644500
H	-2.34476100	6.11673900	-1.81992400
H	-4.56496700	2.52141600	0.40484200
H	-3.99932300	4.71099500	-0.65420700
H	-3.91848500	-0.24903600	-0.99676000
H	-3.92973900	-1.76122300	1.54689300
H	-3.99224300	-6.02090800	0.45135700
H	-2.63776200	-5.29474700	-0.45737300
H	-4.27431000	-5.31719200	-1.16762900
H	-6.21530900	-1.62320800	-0.47732200
H	-6.21176500	-2.50720600	1.04742400
H	-6.96171400	0.60745100	-0.55781300
H	-7.72865800	2.66325400	0.57506900
H	-7.54810000	2.86178500	3.03949100

H	-6.60935700	0.98363000	4.35541100
H	-5.85035900	-1.06726800	3.21878900
H	4.56498400	-2.52155400	0.40496700
H	3.99938300	-4.71107900	-0.65422800
H	2.34483600	-6.11676800	-1.82004400
H	0.08082600	-6.39593900	-2.78207000
H	-1.54958300	-4.52600300	-2.65579400
H	-0.94926100	-2.41002500	-1.61641300
H	3.91823100	0.24911800	-0.99683900
H	3.92956100	1.76133100	1.54679300
H	3.99211500	6.02094100	0.45110200
H	2.63762700	5.29476700	-0.45761000
H	4.27418600	5.31716600	-1.16785400
H	6.21518200	1.62324100	-0.47735800
H	6.21160800	2.50726900	1.04736500
H	6.96142300	-0.60755800	-0.55775100
H	7.72841500	-2.66326000	0.57528300
H	7.54806000	-2.86153100	3.03973900
H	6.60948800	-0.98321800	4.35554900
H	5.85042500	1.06758400	3.21876500

3^{R}MNea

C	-2.79767500	3.03166500	-1.12588500
C	-4.16731000	3.04411300	-1.50999100
O	-5.57699900	-1.06669600	-1.88726500
C	-5.12555900	-0.54835300	-0.87317900
N	-2.22478800	1.85975600	-0.68860900
C	-4.89452400	1.84684800	-1.41127900
H	-5.93858700	1.83501600	-1.69852600
N	-5.34600600	-1.01127300	0.36974900
H	-4.97811200	-0.49051100	1.15158500
C	-4.29348900	0.70595500	-0.95134000
C	-9.96707200	-2.68129300	0.52703400
C	-6.13702800	-2.20352800	0.64527600
H	-5.93720400	-2.89056900	-0.17524000
C	-5.64475200	-2.82039700	1.95464400
H	-5.76234400	-2.10965600	2.77608600
H	-6.21977200	-3.71235800	2.19792300
H	-4.59152600	-3.09516300	1.87473300
C	-4.01869300	5.39383900	-2.04733500
H	-4.46895000	6.31206400	-2.39978200
C	-10.49181900	-4.98659700	-0.01425600
H	-11.21802900	-5.76945300	-0.19235000
C	-4.75702200	4.24384300	-1.97013400

H	-5.80028000	4.22732100	-2.25794500
C	-8.18063200	-4.29585100	0.12477900
H	-7.13285300	-4.54897500	0.05274500
C	-9.11462600	-5.27539600	-0.09917800
H	-8.79239300	-6.27950300	-0.34457500
C	-9.47507100	-0.37870300	1.06145900
H	-9.79979300	0.62672600	1.29817500
C	-8.56800100	-2.96912800	0.44518200
C	-7.63148400	-1.91681300	0.68275900
C	-10.39320100	-1.36852100	0.84038500
H	-11.45557700	-1.16495500	0.89790500
C	-2.06075600	4.23028000	-1.21621900
H	-1.01948300	4.24262500	-0.93568700
C	-8.09587600	-0.65897600	0.98060600
H	-7.39075500	0.14360700	1.15123800
C	-2.66274500	5.37924500	-1.66641900
H	-2.08152600	6.28986500	-1.73034900
C	-10.90441300	-3.71636900	0.29089700
H	-11.95994400	-3.48100600	0.35636600
C	-2.91915000	0.72646200	-0.60396500
C	-2.22266300	-0.52333500	-0.16486600
H	-1.28769500	-0.64701200	-0.70924400
H	-2.84031500	-1.39919400	-0.33699000
H	-1.98188800	-0.47642000	0.89957900
C	2.79900800	3.03250100	1.12620800
C	4.16967900	3.04566000	1.50656100
O	5.57779900	-1.06622000	1.88671400
C	5.12604400	-0.54798700	0.87273600
N	2.22520700	1.86002900	0.69162400
C	4.89684200	1.84836300	1.40743600
H	5.94172500	1.83713500	1.69173700
N	5.34523500	-1.01162000	-0.37014900
H	4.97729500	-0.49085800	-1.15195900
C	4.29462200	0.70676500	0.95091000
C	9.96565600	-2.68330900	-0.52786400
C	6.13576300	-2.20420400	-0.64571600
H	5.93583900	-2.89111700	0.17487700
C	5.64299900	-2.82098400	-1.95492700
H	5.76082900	-2.11043600	-2.77649800
H	6.21751800	-3.71328300	-2.19815800
H	4.58963800	-3.09518300	-1.87480100
C	4.02202200	5.39596200	2.04164700
H	4.47306700	6.31469300	2.39176500
C	10.48964300	-4.98870300	0.01377700

H	11.21560000	-5.77177400	0.19195800
C	4.76041600	4.24605800	1.96362300
H	5.80450800	4.23011800	2.24844200
C	8.17868800	-4.29718500	-0.12521200
H	7.13082500	-4.54992800	-0.05308200
C	9.11235900	-5.27701700	0.09883800
H	8.78979600	-6.28097500	0.34441500
C	9.47440300	-0.38063400	-1.06261900
H	9.79945000	0.62464200	-1.29953500
C	8.56649200	-2.97064600	-0.44584100
C	7.63032000	-1.91803600	-0.68346600
C	10.39221000	-1.37073600	-0.84147600
H	11.45465200	-1.16755400	-0.89911800
C	2.06201500	4.23100400	1.21741900
H	1.01988700	4.24273100	0.94002600
C	8.09512000	-0.66040800	-0.98156100
H	7.39025900	0.14240100	-1.15219100
C	2.66497100	5.38059600	1.66471800
H	2.08366600	6.29110100	1.72948200
C	10.90265700	-3.71866800	-0.29162400
H	11.95826700	-3.48369000	-0.35720400
C	2.91941800	0.72668400	0.60696400
C	2.22231800	-0.52386100	0.17096300
H	1.28697000	-0.64556400	0.71505800
H	2.83942500	-1.39959200	0.34587800
H	1.98232200	-0.47980100	-0.89379400
I	0.00001800	1.86619500	0.00208200

3^{R}MB-I

I	0.00000900	2.06129800	-0.00013500
C	3.10224300	3.03258900	-0.31997900
C	4.49120600	2.85328900	-0.58964800
O	5.63290200	-1.03620900	-1.80728100
C	4.64496100	-0.89028800	-1.09669600
N	2.27538100	1.93983700	-0.32799400
C	4.96584900	1.55636400	-0.84671000
H	6.01338700	1.40029700	-1.06819400
N	3.98030000	-1.91540700	-0.52817400
H	3.26633700	-1.71648400	0.15510400
C	4.10747300	0.48300700	-0.81367000
C	7.65043500	-4.90452400	0.48498700
C	4.40178000	-3.30433200	-0.67618400
H	4.68649200	-3.43962900	-1.71943000
C	3.21495300	-4.21072000	-0.35755200

H	2.89152700	-4.07246900	0.67694900
H	3.49817800	-5.25510200	-0.48479600
H	2.37652700	-3.99307400	-1.02192100
C	4.84266900	5.22876400	-0.33165000
H	5.49613100	6.09086300	-0.33177400
C	5.34453900	3.98161000	-0.58971800
H	6.39630100	3.83273700	-0.79707700
C	2.74962500	0.72886500	-0.55451200
H	2.02192900	-0.07256200	-0.56132600
C	6.84347200	-3.49183200	2.25462800
H	6.94930800	-3.07824300	3.24990600
C	6.56475700	-4.53442800	-0.30063900
C	5.60783700	-3.64479000	0.18213000
C	7.79287300	-4.38381900	1.76681400
H	8.64027000	-4.66601200	2.37868300
C	2.61020500	4.32462900	-0.05575900
H	1.56045900	4.46456100	0.15504100
C	5.75780700	-3.12620000	1.46665700
H	5.02898300	-2.42503200	1.85418300
C	3.46867100	5.39563800	-0.06208500
H	3.08377500	6.38585600	0.14345400
C	-3.10220000	3.03257900	0.31996200
C	-4.49112300	2.85327700	0.58984000
O	-5.63273700	-1.03624100	1.80735400
C	-4.64483100	-0.89032100	1.09671900
N	-2.27534500	1.93981600	0.32778400
C	-4.96574200	1.55634400	0.84690000
H	-6.01323700	1.40027900	1.06858300
N	-3.98024500	-1.91542700	0.52809300
H	-3.26635200	-1.71649000	-0.15525600
C	-4.10738200	0.48298000	0.81364600
C	-7.65068900	-4.90421800	-0.48473400
C	-4.40174000	-3.30435400	0.67608400
H	-4.68633900	-3.43969000	1.71935600
C	-3.21496500	-4.21074200	0.35726900
H	-2.89166000	-4.07244500	-0.67726300
H	-3.49818600	-5.25512800	0.48449200
H	-2.37646000	-3.99313900	1.02155100
C	-4.84260500	5.22876800	0.33200600
H	-5.49606000	6.09087200	0.33227100
C	-5.34444400	3.98160700	0.59010000
H	-6.39617500	3.83273800	0.79762100
C	-2.74957600	0.72883200	0.55427300
H	-2.02190200	-0.07261600	0.56089600

C	-6.84366600	-3.49175200	-2.25453100
H	-6.94952200	-3.07822000	-3.24982900
C	-6.56490300	-4.53421100	0.30079100
C	-5.60790500	-3.64473300	-0.18210800
C	-7.79315100	-4.38358300	-1.76658300
H	-8.64062800	-4.66570600	-2.37837400
C	-2.61019300	4.32463000	0.05573000
H	-1.56048000	4.46455100	-0.15523800
C	-5.75789900	-3.12621100	-1.46666300
H	-5.02901200	-2.42516000	-1.85428200
C	-3.46864900	5.39564500	0.06223100
H	-3.08378200	6.38586800	-0.14333700
H	8.38875300	-5.59298100	0.09316200
H	6.46184500	-4.93362700	-1.30302500
H	-6.46196900	-4.93335700	1.30319500
H	-8.38907000	-5.59255200	-0.09280900

3^{R}Nea-I

I	0.00021400	2.89775100	-0.00020600
C	3.10465000	3.87470300	-0.27042600
C	4.49844000	3.69806300	-0.51574900
O	5.68283400	-0.20012000	-1.65398600
C	4.67551300	-0.05040900	-0.97218000
N	2.28229000	2.77840700	-0.27801500
C	4.98231000	2.40014500	-0.74882600
H	6.03364800	2.24546200	-0.95255700
N	3.99784800	-1.07248700	-0.41314700
H	3.26324200	-0.87077800	0.24691500
C	4.12790000	1.32367800	-0.71514100
C	7.47138400	-4.26484300	0.97388900
C	4.41710300	-2.46076900	-0.55328200
H	4.76286900	-2.56602000	-1.57982100
C	3.19992500	-3.35891800	-0.33371500
H	2.79568900	-3.21239000	0.67068500
H	3.47603500	-4.40745000	-0.43353900
H	2.42120300	-3.13273300	-1.06422100
C	4.83633500	6.07795000	-0.28223600
H	5.48615000	6.94279500	-0.28307600
C	8.18766900	-6.05610900	-0.49621800
H	8.85604200	-6.87705000	-0.72274100
C	5.34718100	4.82984100	-0.51686700
H	6.40257000	4.68267800	-0.70623500
C	6.30384300	-4.68193400	-1.12921700
H	5.52491000	-4.44934200	-1.84084800

C	2.76506600	1.56665000	-0.48075300
H	2.04194200	0.76111500	-0.48814000
C	7.15971000	-5.71926100	-1.40021200
H	7.04642800	-6.28395600	-2.31700300
C	6.78444500	-2.48557000	2.45433000
H	6.90379600	-1.91810200	3.36864900
C	6.42601700	-3.91870400	0.06047800
C	5.56220100	-2.82711800	0.38122100
C	7.62603700	-3.52634500	2.17124600
H	8.42233700	-3.79894200	2.85301900
C	2.60342900	5.16778800	-0.02997000
H	1.55014400	5.30617000	0.16321500
C	5.75556800	-2.14040300	1.55479600
H	5.11149700	-1.30577100	1.79823400
C	3.45764400	6.24222900	-0.03617400
H	3.06557100	7.23328600	0.15090300
C	8.33710900	-5.34175800	0.66319500
H	9.12455100	-5.58915500	1.36499200
C	-3.10425800	3.87435500	0.27054300
C	-4.49795800	3.69758900	0.51629800
O	-5.68189300	-0.20081400	1.65408400
C	-4.67467300	-0.05096000	0.97216300
N	-2.28182000	2.77812100	0.27776200
C	-4.98165800	2.39960600	0.74935200
H	-6.03291300	2.24480200	0.95342000
N	-3.99706800	-1.07295000	0.41286800
H	-3.26274400	-0.87112000	-0.24747500
C	-4.12718700	1.32319100	0.71521200
C	-7.47263700	-4.26322800	-0.97350400
C	-4.41659100	-2.46120100	0.55262000
H	-4.76179500	-2.56684100	1.57930100
C	-3.19980800	-3.35960300	0.33195700
H	-2.79615000	-3.21287800	-0.67264100
H	-3.47620900	-4.40807900	0.43162000
H	-2.42055600	-3.13392100	1.06205600
C	-4.83609900	6.07747900	0.28315900
H	-5.48597100	6.94227900	0.28429800
C	-8.18938800	-6.05429900	0.49661800
H	-8.85816700	-6.87486700	0.72329300
C	-5.34677800	4.82930800	0.51783100
H	-6.40209400	4.68204400	0.70752900
C	-6.30442000	-4.68141100	1.12901200
H	-5.52500500	-4.44945100	1.84032100
C	-2.76444700	1.56629400	0.48044600

H	-2.04123900	0.76083000	0.48753500
C	-7.16080400	-5.71825600	1.40020500
H	-7.04744700	-6.28319800	2.31683400
C	-6.78515600	-2.48421900	-2.45401200
H	-6.90451100	-1.91657400	-3.36822100
C	-6.42665600	-3.91788400	-0.06048800
C	-5.56230200	-2.82679600	-0.38143300
C	-7.62732500	-3.52447000	-2.17069200
H	-8.42409300	-3.79645400	-2.85216300
C	-2.60321300	5.16749500	0.03004600
H	-1.54999900	5.30597900	-0.16344300
C	-5.75569200	-2.13983000	-1.55486200
H	-5.11115400	-1.30560600	-1.79847500
C	-3.45750400	6.24187600	0.03664300
H	-3.06556000	7.23297600	-0.15048100
C	-8.33890100	-5.33965300	-0.66260100
H	-9.12680500	-5.58643700	-1.36409500

3^{L}Phe-I

C	-2.93145200	4.95700900	-1.00970800
C	-1.60710900	4.47838200	-1.06614300
C	-1.31712600	3.15927500	-0.81805500
C	-2.35412900	2.26323000	-0.50089100
C	-3.69607600	2.73942200	-0.43650800
C	-3.95723600	4.10327600	-0.70189400
N	-2.10349500	0.93547300	-0.25722600
C	-3.08308100	0.10334500	0.02755900
C	-4.42306400	0.51330700	0.12773700
C	-4.72187700	1.83240000	-0.10988400
C	-5.44951700	-0.53757900	0.41269600
N	-6.53196200	-0.13514400	1.10268600
O	-5.29642600	-1.68959000	0.01757300
C	-7.67134400	-1.00444800	1.32244200
C	-7.22814500	-2.25489400	2.08774500
C	-8.45713800	-1.27686200	0.02433200
C	-8.82258200	0.00765400	-0.67109500
C	-8.06554700	0.47497100	-1.74532700
C	-8.36490800	1.68880800	-2.35517500
C	-9.43015700	2.45587500	-1.89595000
C	-10.19530900	1.99789200	-0.82738900
C	-9.89208200	0.78392200	-0.22173600
O	-7.92283600	-3.33527300	1.73397900
O	-6.39302600	-2.24085400	2.95827800
C	-7.62049000	-4.55134000	2.44740400

I	-0.00000500	0.00000500	-0.28938800
N	2.10345900	-0.93543700	-0.25726500
C	3.08302200	-0.10327400	0.02756300
C	4.42301200	-0.51321600	0.12774100
C	4.72187200	-1.83229600	-0.10988900
C	3.69610500	-2.73934200	-0.43654500
C	2.35415400	-2.26317600	-0.50095400
C	5.44945000	0.53767900	0.41272100
N	6.53195100	0.13521400	1.10260000
O	5.29627200	1.68973500	0.01775800
C	7.67134700	1.00451900	1.32233000
C	7.22813200	2.25505500	2.08748300
O	7.92296200	3.33537100	1.73380100
C	8.45724700	1.27674600	0.02424800
C	7.62055700	4.55149700	2.44709600
C	8.82264200	-0.00786400	-0.67102500
O	6.39284600	2.24110200	2.95786000
C	8.06563100	-0.47524900	-1.74524200
C	8.36494700	-1.68917000	-2.35494800
C	9.43012700	-2.45625400	-1.89559300
C	10.19525500	-1.99820500	-0.82704400
C	9.89207400	-0.78415200	-0.22153400
C	3.95727100	-4.10318700	-0.70195000
C	2.93150100	-4.95692100	-1.00980900
C	1.60715200	-4.47832000	-1.06628000
C	1.31715400	-3.15921800	-0.81817000
H	-3.13191200	6.00089700	-1.21051300
H	-0.80424100	5.16146100	-1.31019500
H	-0.29922200	2.80231400	-0.86579800
H	-4.98061900	4.45227300	-0.65364600
H	-2.82116200	-0.93238500	0.19380400
H	-5.74237100	2.19237300	-0.07602900
H	-6.56420800	0.79586400	1.48310700
H	-8.32810700	-0.47173600	2.01367300
H	-9.35463300	-1.83603200	0.28510900
H	-7.85504800	-1.90148000	-0.63273400
H	-7.23354800	-0.11853400	-2.10468100
H	-7.76709500	2.03378900	-3.18974500
H	-9.66730800	3.39956700	-2.37075800
H	-11.03185200	2.58473100	-0.46878300
H	-10.49428600	0.43263800	0.60835600
H	-8.28266500	-5.30457300	2.02980200
H	-6.57940400	-4.83000300	2.29005600
H	-7.81163000	-4.42370900	3.51212900

H	2.82109500	0.93244500	0.19383300
H	5.74237200	-2.19225300	-0.07600800
H	6.56419800	-0.79579800	1.48301300
H	8.32802300	0.47184700	2.01367000
H	9.35476400	1.83588200	0.28503000
H	7.85525000	1.90135000	-0.63291200
H	8.28276400	5.30469600	2.02948300
H	6.57948500	4.83015300	2.28965300
H	7.81162000	4.42395700	3.51184900
H	7.23368500	0.11826400	-2.10470200
H	7.76715200	-2.03419900	-3.18951100
H	9.66723800	-3.40000900	-2.37029800
H	11.03174500	-2.58505600	-0.46833400
H	10.49426600	-0.43282400	0.60854700
H	4.98065200	-4.45219100	-0.65368900
H	3.13199200	-6.00080100	-1.21062200
H	0.80430400	-5.16140400	-1.31037400
H	0.29925400	-2.80224300	-0.86593800

Reference

[1] Carlsson A-C C, Mehmeti K, Uhrbom M, et al. Substituent Effects on the [N–I–N]$^+$ Halogen Bond [J]. *J. Am. Chem. Soc.,* **2016**, 138(31): 9853-63.

[2] Ward J S, Frontera A, Rissanen K. Nucleophilic iodonium interactions (NIIs) in 2-coordinate iodine(i) and silver(i) complexes [J]. *Chem. Comm.* **2021**, 57(41): 5094-7.

[3] M. J. Frisch, G. W. Trucks, H. B. Schlegel, G. E. Scuseria, M. A. Robb, J. R. Cheeseman, G. Scalmani, V. Barone, G. A. Petersson, H. Nakatsuji, X. Li, M. Caricato, A. V. Marenich, J. Bloino, B. G. Janesko, R. Gomperts, B. Mennucci, H. P. Hratchian, J. V. Ortiz, A. F. Izmaylov, J. L. Sonnenberg, D. Williams-Young, F. Ding, F. Lipparini, F. Egidi, J. Goings, B. Peng, A. Petrone, T. Henderson, D. Ranasinghe, V. G. Zakrzewski, J. Gao, N. Rega, G. Zheng, W. Liang, M. Hada, M. Ehara, K. Toyota, R. Fukuda, J. Hasegawa, M. Ishida, T. Nakajima, Y. Honda, O. Kitao, H. Nakai, T. Vreven, K. Throssell, J. A. Montgomery, Jr., J. E. Peralta, F. Ogliaro, M. J. Bearpark, J. J. Heyd, E. N. Brothers, K. N. Kudin, V. N. Staroverov, T. A. Keith, R. Kobayashi, J. Normand, K. Raghavachari, A. P. Rendell, J. C. Burant, S. S. Iyengar, J. Tomasi, M. Cossi, J. M. Millam, M. Klene, C. Adamo, R. Cammi, J. W. Ochterski, R. L. Martin, K. Morokuma, O. Farkas, J. B. Foresman, and D. J. Fox, Gaussian, Inc., Wallingford CT, 2016.
